# The pause-initiation limit restricts transcription activation in human cells

**DOI:** 10.1038/s41467-019-11536-8

**Published:** 2019-08-09

**Authors:** Saskia Gressel, Björn Schwalb, Patrick Cramer

**Affiliations:** Department of Molecular Biology, Max-Planck-Institute for Biophysical Chemistry, Am Faßberg 11, 37077 Göttingen, Germany

**Keywords:** Computational models, Gene regulation, RNA sequencing, Transcription, Time series

## Abstract

Eukaryotic gene transcription is often controlled at the level of RNA polymerase II (Pol II) pausing in the promoter-proximal region. Pausing Pol II limits the frequency of transcription initiation (‘pause-initiation limit’), predicting that the pause duration must be decreased for transcriptional activation. To test this prediction, we conduct a genome-wide kinetic analysis of the heat shock response in human cells. We show that the pause-initiation limit restricts transcriptional activation at most genes. Gene activation generally requires the activity of the P-TEFb kinase CDK9, which decreases the duration of Pol II pausing and thereby enables an increase in the productive initiation frequency. The transcription of enhancer elements is generally not pause limited and can be activated without CDK9 activity. Our results define the kinetics of Pol II transcriptional regulation in human cells at all gene classes during a natural transcription response.

## Abstract (web summary)

Gene activation requires an increase of successful initiation events. Here, by employing a genome-wide kinetic analysis of transcription, the authors showed that gene activation generally requires a decrease in RNA Polymerase II (Pol II) promoter-proximal pausing while transcription of enhancer elements is not limited by Pol II pausing.

## Introduction

Gene transcription is regulated at multiple levels^1–3^. A critical point of transcription regulation in human cells is the phase of early elongation by RNA polymerase (Pol) II^4–6^ (reviewed in ref. ^7^). After Pol II escapes from the promoter, it often pauses in the promoter-proximal region, and this represents a major regulatory step at protein-coding genes^8,9^. Paused Pol II is stabilized by the factors DSIF^10^ and NELF^11^, and is released into active elongation by the CDK9-containing kinase complex P-TEFb^12,13^. Recent studies revealed structures of the Pol II elongation complex in the paused and activated state, and provided mechanistic insights into the P-TEFb dependent switch to active elongation^14,15^. Pol II pauses also at noncoding genes that produce enhancer RNAs^16^, upstream antisense RNAs (uaRNAs)^17^, and other long noncoding RNAs^18,19^. However, whether and to which extent pausing can restrict the transcriptional output at different gene classes was not quantified and compared in vivo.

Most available studies estimated the degree of Pol II pausing as the relative ratio of Pol II occupancy in the promoter-proximal region and the gene body, which has been termed the traveling ratio^20^, or the pausing index^21,22^. Pol II occupancy can be mapped using DNA by chromatin immunoprecipitation (IP) assays^23^, and along nascent RNA transcripts by nuclear run-on assays^8,24^ or by native elongating transcript sequencing (NET-seq)^25,26^. The latter methods are powerful tools to locate engaged Pol II in a strand-specific manner and at high resolution. Although, Pol II occupancy depends on pausing it does not directly relate to the kinetics of pausing^27^. This is because the Pol II occupancy signal at a given time depends on the number of polymerases and their speed, and cannot be used in isolation to distinguish between these two. Indeed, when a Pol II occupancy peak increases, this can be due to an increase in the number of pausing polymerases or due to an increase in the duration of pausing, or both.

**Fig. 1:**
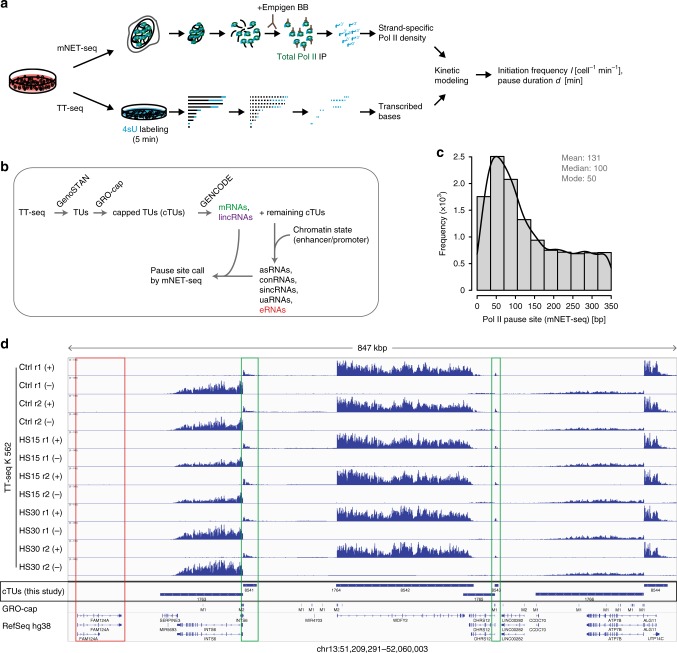
Multiomics analysis and annotation of different gene classes in human K562 cells. **a** Experimental schematic of multiomics approach. Human K562 cells were subjected to TT-seq to estimate the productive initiation frequency *I* [cell^−1^min^−^^1^]. By combining TT-seq data with mNET-seq the pause duration *d* [min] is calculated (“Methods”)^28^. **b** Schematic overview of capped transcription unit (cTU) annotation in K562 cells. First, TT-seq data (*n* = 6) were subjected to the genomic segmentation algorithm GenoSTAN^42^. The transcription start sites (TSSs) were further refined by published GRO-cap data generated in K562 cells^43^. To call promoter-proximal pause sites mNET-seq data (*n* = 6) generated in K562 cells were used. (Supplementary Table 1, Supplementary Fig. 1). **c** Distribution of pause site distance [bp] for 12,160 investigated cTUs measured by mNET-seq depicted as a histogram with respect to GRO-cap refined TSS (mean 131 [bp], median 100 [bp], and mode 50 [bp]). **d** Genome browser view of a 847-kbp region located in chromosome 13 (chr13: 51,209,291–52,060,003) visualized with the Integrative Genomics Viewer (IGV, version 2.4.10; human hg38)^81^. From top to bottom, tracks represent: TT-seq coverage in K562 cells (*n* = 6) on respective plus (+) or minus (−) strand, GRO-cap data^43^, new cTU annotation, RefSeq GRCh38 annotation. cTUs missing in the RefSeq GRCh38 annotation are highlighted in a green. RefSeq transcript not present in K562 cells is highlighted in red

As a consequence, the time polymerases reside in a pause window can only be estimated by factoring in the number of polymerases that pass through the pause window. This number we have called the productive initiation frequency *I*, which is defined as the number of Pol II enzymes that initiated at the promoter and were successfully released from the pause window into productive elongation^28^. *I* is therefore independent of a putative unknown fraction of polymerases that may be terminating in the pause window. The productive initiation frequency *I* can be measured by transient transcriptome sequencing (TT-seq), which is a sensitive genome-wide assay that captures RNA that is newly transcribed within 5 min of metabolic labeling with 4-thiouridine (4sU)^29^. After labeling, RNA is fragmented, and only newly synthesized RNA fragments are purified and sequenced. When TT-seq is combined with mammalian NET-seq (mNET-seq), the pause duration *d* can also be obtained^28^ (Fig. 1a). This multiomics approach provided evidence for a “pause-initiation limit” that restricts the productive initiation frequency at a given pause duration^28^. Such a limit was predicted based on steric considerations^27^, and an independent study also concluded that pausing can impair initiation^30^.

These studies suggested that transcriptional activation of genes near the pause-initiation limit must be enabled by a decrease in the duration of Pol II pausing, but this was not investigated by a genome-wide kinetic analysis before. In order to achieve this, we first defined transcription kinetics for protein-coding and noncoding genes in steady state in human cells. We then used our multiomics approach to follow changes of productive initiation frequency *I* and pause duration *d* in a quantitative manner during the dynamic transcriptional response to heat shock over time. The heat shock response in the human hematopoietic cell line K562 was chosen because it provides a well-established model system^31–36^, and it involves global transcriptional mechanisms that are conserved across species (reviewed in ref. ^37^). It was also shown that recruitment of P-TEFb is critical for Pol II release upon heat shock from selected model genes^38–41^.

Results presented here show that transcriptional activation of protein-coding genes involves an increase in the productive initiation frequency, as expected, and is indeed restricted by the pause-initiation limit, as predicted. We also show that inhibition of the P-TEFb kinase CDK9 impairs full activation upon heat shock, confirming that a decrease in Pol II pause duration is a critical step in gene activation. In contrast, we find that transcription of enhancers, which also involves Pol II pausing^16^, is generally not restricted by the pause-initiation limit. Instead, enhancer transcription can be strongly upregulated and downregulated by changes of the productive initiation frequency alone, even when the P-TEFb kinase CDK9 is inhibited. Taken together, we describe the kinetic basis for gene regulatory strategies underlying a transcription response in human cells, and demonstrate in a quantitative manner that promoter-proximal Pol II pausing defines a pause-initiation limit that restricts gene activation by limiting the increase in productive initiation frequency.

## Results

### Multiomics analysis and annotation of transcription units

**Fig. 2:**
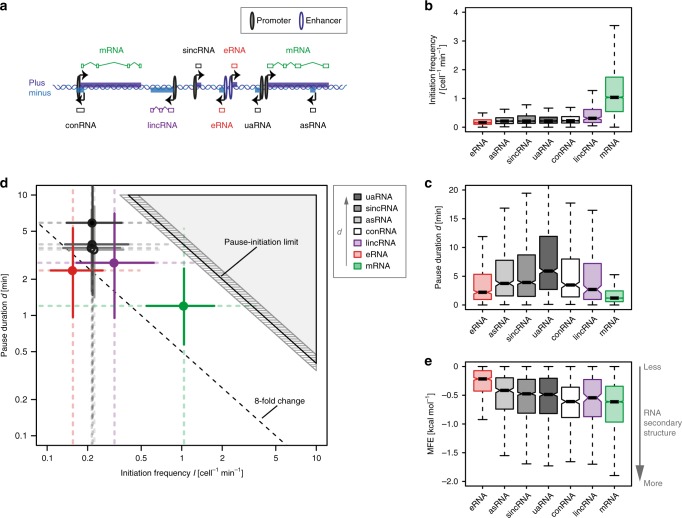
Transcription kinetics of different gene classes in steady state. **a** Schematic representation of seven major transcript classes annotated in this study: 604 eRNAs (in light red), 471 asRNAs, 1314 sincRNAs, 965 uaRNAs, 445 conRNAs (in different shades of gray), 209 lincRNAs (in purple), and 6355 mRNAs (in green), depicted on plus (dark blue) or minus (light blue) strand (“Methods”). **b** Boxplots of productive initiation frequency *I* [cell^−1^min^−1^] for transcript classes defined in **a**. Black bars represent medians, boxes span from upper to lower quartiles, and whiskers represent 1.5 times the interquartile range. **c** Boxplots of pause duration *d* [min]. **d** Plot shows the median productive initiation frequency *I* [cell^−1^min^−1^] depicted against the median pause duration *d* [min] for all transcript classes (circles) in log scale. The two solid perpendicular lines define the interquartile range, and the dotted whiskers represent 1.5 times the interquartile range of the respective estimates for the entire transcript class. The gray shaded area depicts impossible combinations of *I* and *d*^27,28^. Striped area shows confidence intervals of the pause-initiation limit. The dotted line defines an eightfold possible fold change until a gene would be restricted by the pause-initiation limit. **e** Boxplots of minimum free energy MFE [kcal mol^−1^]. MFE was calculated in a window of [−15,−65] bp upstream of the pause site to predict RNA secondary structure^79^ for each transcript in a transcript class

We first aimed at defining genome-wide Pol II kinetic parameters during steady-state conditions. To obtain the productive initiation frequency *I* and the promoter-proximal pause duration *d*^28^, we carried out TT-seq and mNET-seq of total Pol II (with Empigen BB) in human K562 cells (Fig. 1a). We generated TT-seq data for two independent biological replicates after 5 min of metabolic labeling with 4sU (Spearman correlation rho = 1.00) as well as mNET-seq data for two independent biological replicates (Spearman correlation rho = 0.99) (Supplementary Table 1; Supplementary Fig. 1). We then used the TT-seq data to create a genome-wide transcription unit (TU) annotation with the segmentation algorithm GenoSTAN^42^ (Fig. 1b). As a strategy for identifying accurate transcription start sites (TSSs) for the TT-seq derived TUs, we used published GRO-cap data^43^, which recovers nascent RNAs with 5′ caps of transcriptionally engaged Pol II. To be eligible for further analysis, annotated TUs needed a GRO-cap signal in a window of 250 bp around the GenoSTAN-derived start site, and an expression of >5 reads per base in the sum of both replicates of the TT-seq signal. We then sorted each capped TU (cTU) into one of the following seven classes using GENCODE annotation, respective location, and chromatin state annotation^42^ for enhancer and promoter classification (Fig. 2a; “Methods”): protein-coding (m), long intergenic noncoding (linc), antisense (as), convergent (con), upstream antisense (ua), short intergenic noncoding (sinc), and putative enhancer (e) RNAs.

Subsequently, we used the mNET-seq data to extract the position of paused polymerases for all cTUs in each class that showed mNET-seq signal peaks above background (“Methods”). The called pause sites were distributed around a maximum located ~50 bp downstream of the TSS (Fig. 1c), in contrast to the pause sites that were previously derived based on the TSS annotation from RefSeq, which were located ~30 bp further downstream (Supplementary Fig. 5c)^28^. This agrees well with recently published data in K562 cells^44^. We did not observe any substantial differences in the positions of called pause sites among the different classes. This resulted in a total of 10363 expressed cTUs, for which a pause site call was successful, encoding 604 eRNA, 471 asRNA, 1314 sincRNA, 965 uaRNA, 445 conRNA, 209 lincRNA, and 6355 mRNAs (Figs. 1b–d and 2a). Below we will refer to these cTUs simply as “genes” or with the respective RNA transcript class they give rise to.

### Transcription kinetics differ between gene classes

We next used TT-seq and mNET-seq data in combination with our previously described kinetic modeling^28^ to derive estimates of *I* and *d* for all annotated genes (Fig. 2a; “Methods”). We observed a reciprocal behavior of *I* and *d* for all classes of genes except enhancers (Fig. 2b–d). When *I* was high, *d* was generally low, and the other way around, consistent with an anticorrelation between these two parameters^28,30^. Protein coding and lincRNAs showed the highest *I* value (with median initiation events of 1 and 0.3 cell^−1^min^−1^) and the lowest *d* values (median 1 and 2.7 min), consistent with high expression levels (Fig. 2b, d; Supplementary Figs. 2a and 6a, b). On average, lincRNAs show significantly longer pausing compared with mRNAs (*p*-value < 2.2 × 10^−16^, Wilcoxon rank sum test) (Fig. 2c, d; Supplementary Fig. 2a), in contrast to a recent study^45^. Thus, genes encoding lincRNAs initiate on average about half as frequently as protein-coding genes, and smaller mNET-seq peaks for these genes indicate longer pause durations, which is counterintuitive but clearly revealed by the kinetic analysis (Supplementary Fig. 6a, b). Generally, all noncoding RNA classes (except eRNAs) showed high *d* values, with a median ranging around 4–6 min, explaining their low levels of expression. The low pause durations at enhancers might be explained by the fact that eRNAs do generally not adopt stable secondary structure in the nascent RNA exiting from the Pol (Fig. 2e; Supplementary Fig. 6c), which is associated with pausing^28^. However, other chromatin features and factor availability at different loci might contribute to pausing as well (Supplementary Fig. 2b). A particularly long pause duration was observed for uaRNAs (median 6 min), and this could impair initiation events in the noncoding direction of bidirectional promoters (Fig. 2c; Supplementary Fig. 6d).

### Transcription kinetics of a natural transcription response

**Fig. 3:**
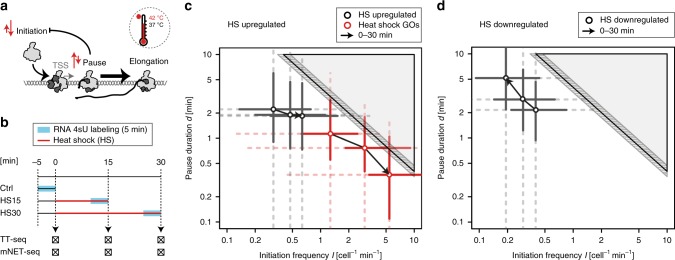
Regulation of transcription upon heat shock timecourse in human K562 cells. **a** Illustration of the regulatory changes (red arrows) at a model gene upon heat shock. Pol II is depicted in silver, decorated with initiation, pausing or elongation factors (dark gray). **b** Experimental setup of the heat shock (HS) timecourse (0–30 min at 42 °C) in human K562 cells. TT-seq and mNET-seq experiments were performed in two independent biological samples (Supplementary Table 1; Supplementary Fig. 1). **c** Pause-initiation trajectories upon heat shock timecourse. Shown are 706 significantly upregulated cTUs (in gray), and 33 significantly upregulated RefSeq-TUs (in red) of overrepresented GO categories linked to heat shock (unfolded protein binding, regulation of cellular response to heat, response to unfolded protein, and chaperone binding) (Supplementary Fig. 7). Median estimates per time point (0, 15, and 30 min) are depicted as circles. The two solid perpendicular lines define the interquartile range, and the dotted whiskers represent 1.5 times the interquartile range of the respective estimates for the entire transcript class. The gray shaded area depicts impossible combinations of *I* and *d*^27,28^. Striped area shows confidence intervals of the pause-initiation limit. **d** Pause-initiation trajectories as in **c** upon heat shock timecourse of 1907 significantly downregulated cTUs (in gray)

We next investigated how transcription activation kinetics change upon response to heat shock (Fig. 3). The optimal time points were determined by quantitative reverse transcription PCR (RT-qPCR) of the major cell stress protein HSPA1A (human Hsp70) and cell viability assays (Supplementary Fig. 3). We performed TT-seq, RNA-seq, and mNET-seq in K562 cells that were maintained under optimal growth conditions at 37 °C (control), or placed in a 42 °C water bath for 15 or 30 min (heat shock) (Fig. 3b). TT-seq, RNA-seq, and mNET-seq libraries were prepared in two biological replicates that were highly reproducible (TT-seq: rho > 0.99 for all time points; RNA-seq: rho > 0.99 for all time points; and mNET-seq: rho > 0.99 for all time points) (Supplementary Fig. 1; Supplementary Table 1). In order to capture global changes in transcription profiles, we used a spike-in normalization strategy (“Methods”)^29^. This revealed that 899 genes were significantly upregulated, whereas 2614 genes were downregulated after 30 min of heat shock (Supplementary Fig. 4a). To normalize the respective mNET-seq signals in the heat shock conditions versus the control, we identified 3416 genes that were unchanged in their TT-seq signal, and globally calibrated the mNET-seq data to show no change on these genes during the heat shock response (“Methods”).

### The pause-initiation limit restricts transcription activation

Next, we calculated the productive initiation frequency *I* and the pause duration *d* for all genes after 15 min and after 30 min of heat shock. Our results show that upon heat shock, activated genes generally show increased *I* and decreased *d* (Fig. 3c; Supplementary Fig. 5f), suggesting that gene activation requires a decrease in pause duration, which in turn allows for higher productive initiation frequencies. This behavior is even more evident at protein-coding genes linked to heat shock (Fig. 3c). This can be exemplified at the HSPA1A (HSP70) gene, which is a prominent model gene for the heat shock response (Supplementary Fig. 4b). The pause duration *d* of HSPA1A changes from ∼30 min at steady state to ∼0 min upon 30 min of heat shock which agrees well with literature estimates^46^. Consistent with an anticorrelation between *d* and *I*, *I* increased upon heat shock to 87 productive initiation events (cell^−1^min^−1^). The results also revealed that downregulated genes exhibited decreased *I* and increased *d* (Fig. 3d; Supplementary Fig. 5g). Thus, this multiomics analysis reveals that the pause-initiation limit restricts gene regulation at genes which are located close to the limit in steady state. The use of TT-seq in this respect is critical because it directly monitors RNA synthesis activity and productive initiation frequency (see also Supplementary Note 1; Supplementary Fig. 6e).

### CDK9 activity lowers the pause-initiation limit

**Fig. 4:**
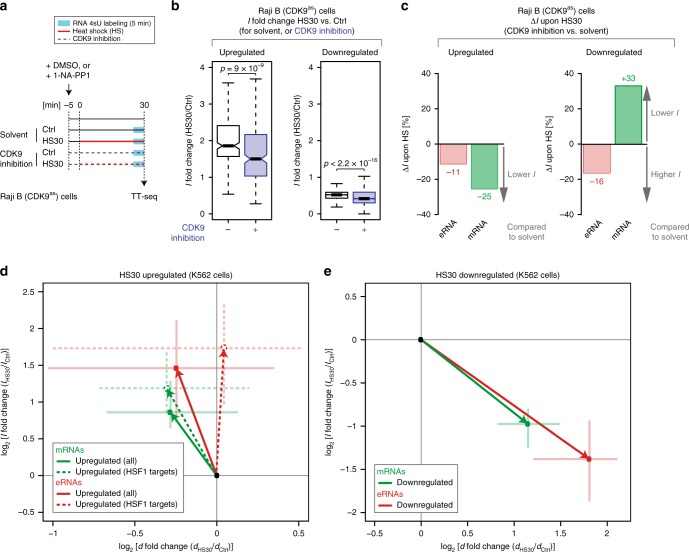
Enhancer transcription is generally not pause limited and less dependent on CDK9. **a** Experimental setup. The adenine analog 1-NA-PP1 allows for rapid and highly specific inhibition of analog-sensitive CDK9^82^ in CRISPR-Cas9 engineered human Raji B (CDK9^as^) cells^28^ (Supplementary Fig. 8b–d). CDK9 was inhibited using 5-µM 1-NA-PP1 (CDK9 inhibition) in combination with heat shock at 42 °C for 30 min in human Raji B (CDK9^as^) cells. DMSO was used as solvent control. **b** Boxplots of productive initiation frequency *I* fold change before (in white) and after CDK9 kinase inhibition (in dark blue). Shown are 241 significantly upregulated genes (left boxplot), and 2795 significantly downregulated genes (right boxplot) annotated in Raji B (CDK9^as^) cells (Supplementary Fig. 9). **c** Δ*I* upon upregulation or downregulation between heat shock with CDK9 inhibition (CDK9 inhibited HS30) and heat shock with solvent control (Solvent HS30) is shown in percent [%]. Left: bar plot comparing productive initiation frequency *I* change (Δ*I*) with and without CDK9 inhibition for 92 significantly upregulated mRNAs (in green), and 54 significantly upregulated eRNAs (in red) annotated in Raji B (CDK9^as^) cells. Right: bar plot comparing productive initiation frequency *I* change (Δ*I*) with and without CDK9 inhibition for 2210 significantly downregulated mRNAs (in green), and 223 significantly downregulated eRNAs annotated in Raji B (CDK9^as^) cells. **d** Log2 fold change of pause duration *d* and initiation frequency *I* for 336 significantly upregulated mRNAs (green solid line), and 67 significantly upregulated eRNAs (red solid line) in K562 cells upon 30 min of heat shock (HS30) (Supplementary Fig. 4a). Dashed lines represent HSF1 driven (“Methods”) subsets of 91 mRNAs and 20 eRNAs. **e** Log2 fold change of pause duration *d* and initiation frequency *I* for 1101 significantly downregulated mRNAs (in green), and 99 significantly downregulated eRNAs (in red) in K562 cells upon 30 min of heat shock (HS30) (Supplementary Fig. 4a)

Our kinetic modeling revealed that the activation of genes restricted by the pause-initiation limit requires a decrease in the pause duration. To corroborate this, we utilized the highly specific and rapid inhibition of an analog-sensitive CDK9 (CDK9^as^) Raji B cell line using the bulky ATP analog 1-NA-PP1^28^ (Fig. 4a). Raji B and K562 cell lines are predicted to show a conserved response to heat shock with respect to timing of HSPA1A upregulation, cell viability, and GO terms of upregulated or downregulated TUs (Supplementary Figs. 3 and 7). We generated TT-seq data for two independent biological replicates after 5 min of metabolic labeling with 4sU to measure changes in *I* upon specific inhibition of the P-TEFb kinase CDK9 prior and during heat shock (Supplementary Table 1). TT-seq data were highly reproducible (Spearman correlation rho = 1.00) (Supplementary Fig. 8a) and CDK9 kinase inhibition was very rapid (Supplementary Fig. 8b–d). We again annotated TUs genome wide with GenoSTAN (Supplementary Fig. 9; “Methods”), and this resulted in 6990 mRNAs, 3451 eRNAs, 243 lincRNAs, 1398 asRNAs, 326 conRNAs, 565 uaRNAs, and 3479 sincRNAs.

We now tested our hypothesis by investigating the changes in *I* upon heat shock after CDK9 inhibition (Fig. 4). We derived estimates of the productive initiation frequency *I* for all 12958 expressed, non-ambiguously classified genes after spike-in normalization (“Methods”). Changes in *I* for upregulated genes confirmed a strong dependence of transcription activation on CDK9 kinase activity. Genes were significantly less inducible during heat shock when CDK9 was inhibited (*p*-value = 9 × 10^−9^, Wilcoxon rank sum test), confirming an obligatory decrease in pause duration for upregulation of the productive initiation frequency (Fig. 4b, left). Productive initiation events of genes encoding mRNAs decreased to 75% after CDK9 inhibition (Fig. 4c, left). Downregulation of genes was overall stronger upon CDK9 inhibition (*p*-value < 2.2 × 10^−16^, Wilcoxon rank sum test) (Fig. 4b, right), indicating less possible initiation events (33% for mRNAs) due to even longer pause durations compared with downregulation upon heat shock alone (Fig. 4c, right). Thus, CDK9 activity lowers the pause duration to allow for high gene activation that is restricted by the pause-initiation limit.

### Enhancer transcription is generally not pause limited

Amongst all seven gene classes, enhancers showed the greatest distance from the pause-initiation limit at steady state in K562 cells, thus forming a notable exception from other gene classes (Fig. 2d). Enhancers showed a median pause duration of 2.2 min and a median productive initiation frequency of only one initiation event every 6–7 min. This indicated that enhancers were generally not restricted by the pause-initiation limit, i.e., *I* could increase several fold without any change in pause duration, until the pause-initiation limit would be reached. Indeed, upon heat shock, the productive initiation frequency *I* for enhancers was increased 1.5-fold more than for mRNAs given the same change in pause duration *d* (Fig. 4d, solid arrows). This difference is even stronger when comparing heat shock factor 1 (HSF1) targeted mRNAs and eRNAs (Fig. 4d, dotted arrows). HSF1 is a major activator in heat shock induced transcription upregulation^47^. HSF1 driven eRNAs can be activated without a change in pause duration, while HSF1 driven mRNAs still require a shortening of the pause duration.

Another implication of this exceptional kinetic behavior is that enhancer transcription can generally only be reduced by a strong increase (>8-fold, Fig. 2d) of the pause duration *d*. However, downregulation of enhancer transcription is in line with the general observation of prolonged pausing to inhibit new initiation events, as is the case for mRNA synthesis (Fig. 4e). In conclusion, enhancers differ from protein-coding genes, because their productive initiation frequency appears generally not to be restricted by the pause duration.

### Enhancer transcription is less dependent on CDK9

Although enhancer transcription is generally not limited by pausing in K562 cells at steady state (Fig. 2d), it remained unclear whether enhancer transcription is controlled by P-TEFb. Using mNET-seq data in Raji B (CDK9^as^) cells^28^ we found that the median pause duration of all transcript classes and the exceptional role of eRNAs is conserved in unperturbed Raji B cells (Supplementary Fig. 10a). We confirmed this also in the context of the upregulation of enhancer transcription upon heat shock in K562 cells. Enhancers showed higher initiation frequency fold changes provided the same fold change in pause duration as found for protein-coding genes (Fig. 4d). Consistent with the results in K562, activation of enhancer transcription in Raji cells was only reduced by 11% upon CDK9 inhibition (Fig. 4c; Supplementary Fig. 10c).

This shows that enhancer transcription can be activated even when CDK9 is inhibited. The overall behavior of impaired activation after CDK9 inhibition for all gene classes resembles the pause durations calculated for Raji cells^28^ and strongly supports our estimates (Fig. 2d; Supplementary Fig. 10b). Surprisingly, downregulated enhancers were not repressed by inhibition of CDK9, consistent with our assumption that higher pause durations do not cause lower productive initiation frequencies at enhancers (Fig. 4c, right; Supplementary Fig. 10d). Taken together, enhancer transcription and thus eRNA synthesis can be upregulated and downregulated to a large extent without changes in pause duration.

## Discussion

To understand genome function, the regulatory steps of gene transcription must be defined and it must be analyzed under which conditions they become rate limiting. A rate-limiting step may be defined as the slowest molecular transition in the process that limits the overall progression and the transcriptional output (reviewed in refs. ^2,48^). We recently showed that prolonged promoter-proximal pausing of Pol II impairs new initiation, and thus reduces the amount of mRNA synthesized per time (“pause-initiation limit”)^28^. Others have also provided evidence that pausing controls initiation^30^. However, changes in the kinetics of Pol II initiation and pausing have not been quantified genome-wide during a transcription response, which is required as definitive evidence that natural gene regulation is controlled by pausing kinetics.

Here we quantified transcription kinetics of protein-coding and noncoding genes in steady state and during the dynamic transcriptional response of human cells to heat shock. To this end, we annotated protein-coding RNAs (mRNAs), and six major noncoding transcript classes, i.e., lincRNAs, asRNAs, eRNAs, uaRNAs, conRNAs, and sincRNAs in human hematopoietic cell lines (K562 and Raji B) (Fig. 1; Supplementary Fig. 9a). We then used a multiomics approach to follow changes in productive initiation frequency *I* and pause duration *d* in a quantitative manner.

In cells at steady state, we observed a reciprocal behavior of *I* and *d* for genes encoding all transcript classes except eRNAs. Protein coding and lincRNAs were among the classes with the shortest pause durations, consistent with their high transcription levels. The longest median pause duration was observed for uaRNAs, presumably impairing initiation events in the noncoding direction of bidirectional promoters (Fig. 2). So far, it was proposed that transcriptional regulation upon heat shock is coordinated at the single step of promoter-proximal pause release^36,49^. Here we could show that this holds true for genes that are close to the pause-initiation limit such as protein-coding genes (Fig. 3). Using the transcriptional response to heat shock, we show that upregulation of the productive initiation frequency is restricted by the P-TEFb kinase CDK9. Enhancers form a notable exception to this rule, because changes in pause duration do not cause changes in eRNA production during heat shock (Fig. 4; Supplementary Fig. 10c, d).

More generally, upregulation of a gene requires an increase in initiation frequency, which leads to a higher number of polymerases loaded onto the gene, and a higher amount of RNA synthesis over time. At genes that are at the pause-initiation limit, pausing limits initiation, and a decrease in pause duration is required for upregulation of transcription by allowing for higher initiation frequencies. This is apparently often the case at protein-coding genes. In contrast, upregulation of enhancer transcription is often possible without changes in pause duration because enhancers are generally not pause limited. These observations lead to the following hypothetical model of gene activation. Gene activation would start with an upregulation of enhancer transcription by an increase in the frequency of initiation from enhancer regions. This would go along with a decrease in pause duration at the protein-coding target genes, which in turn allows for an increase of the productive initiation frequency at these genes. These mechanisms however rely on the availability of polymerases and transcription factors, and assume that transcription is generally processive.

Provided the critical role of decreasing the pause duration for gene activation, the mechanisms of Pol pause release should be studied further in the future. Note that our model holds true independent of the percentage of premature termination that might occur before the promoter-proximal pause site as it quantifies the effective pause between two initiation events that successfully lead to productive elongation. A role in pause release upon heat shock has been reported for the following factors: the TFIIH-associated kinase CDK7^50–52^, the elongation factor TFIIS^53,54^, the DNA-PK kinase, the ATM kinase, the 7SK snRNP recruitment factor TRIM28/KAP1, the pause stabilizing factor GRINL1A/GDOWN1^55,56^, and the CTD phosphatase FCP1^57^. Methods developed here and elsewhere^28^ can now be used to study the kinetics underlying the mechanisms of P-TEFb delivery and activation in a quantitative and genome-wide manner, ultimately unraveling the nature of gene regulation in metazoan cells.

## Methods

### Cell culture

Human K562 erythroleukemia cells were obtained from DSMZ (Cat. # ACC-10). Human Raji Burkitt’s (B) lymphoma (CDK9^as^) cells carry homozygous mutation of phenylalanine (F) 103 to alanine (A) at the CDK9 gene loci and were generated using the CRISPR-Cas9 system^28^. K562 and Raji B (CDK9^as^) cells were cultured antibiotic free in accordance with the DSMZ cell culture standards in RPMI 1640 medium (Thermo Fisher Scientific) containing 10% heat inactivated fetal bovine serum (Thermo Fisher Scientific), and 1× GlutaMAX supplement (Thermo Fisher Scientific) at 37 °C in a humidified 5% CO_2_ incubator. Both cell lines used in this study display the phenotypic properties, including morphology and proliferation rate, that have been described in the literature. Cells were verified to be free of mycoplasma contamination using Plasmo Test Mycoplasma Detection Kit (InvivoGen). Biological replicates were cultured independently.

### Heat shock treatment

To avoid transcriptional changes by freshly added growth medium, fresh growth medium was added ∼24 h prior to heat shock treatments. Heat shock treatments of K562 or Raji B (CDK9^as^) cells were performed in T175 flasks in a volume of 50 mL at 0.6 × 10^6^ cells mL^−1^ in a water bath (LAUDA, Aqualine AL12) at 42 °C. Temperature was monitored by thermometer. It took 5 min until the cell suspension reached 42 °C. For RT-qPCR and cell viability assessment, cells were treated for a timecourse of 0–75 min. For TT-seq, RNA-seq and mNET-seq experiments, cells were treated for 0 (Ctrl), 15 (HS15), or 30 min (HS30).

### Cell viability assessment by trypan blue exclusion test

Cell viability levels were evaluated by the trypan blue exclusion method as described by Strober^58^. Cell viability assessment was performed with two biological replicates. Briefly, 5 × 10^5^ cells were treated as above for 0, 15, 30, 45, 60, or 75 min. Cells were pelleted at 200 × *g* for 5 min and resuspended in 1-mL DPBS prior to counting. Equal volumes of cell suspension and 0.4% trypan blue solution (Sigma-Aldrich) were mixed and incubated for 2 min. The solution was applied to a hemacytometer and viable cells were counted using light microscopy. For each treatment time, the cell count was duplicated (dilution factor for trypan blue) and the average value was obtained. Cell viability was calculated as the ratio of viable cells upon heat shock (15–75 min) to viable cells of control (0 min).

### Total RNA isolation and RT-qPCR

K562 or Raji B (CDK9^as^) cells were treated as above for 0–75 min. For each time point and biological replicate (*n* = 2), 5 × 10^6^ cells were harvested at 3000 × *g* for 2 min. Total RNA was isolated with QIAzol (QIAGEN) according to the manufacturer’s instructions except for the addition of 10 ng RNA spike-in mix^29^ together with QIAzol. To remove possible genomic DNA contamination, isolated RNA (10 µg) was treated with TURBO DNase (Thermo Fisher Scientific) according to the manufacturer’s instructions. For reverse transcription (RT), random hexamer priming (5′-d(NNNNNN)-3′, N = G, A, T, or C) was used according to the manufacturer’s instructions. Briefly, 1 µg of DNase-treated RNA, random hexamer primers (final concentration of 5 ng/µL), and dNTPs mix (final concentration of 0.5 mM) were mixed and incubated at 65 °C for 5 min. Subsequently, Maxima H Minus reverse transcriptase (RT) (final concentration of 200 U) and 5 × Maxima RT buffer (Thermo Fisher Scientific) were added (+RT reaction). For DNA contamination control, cDNA synthesis without RT (−RT reaction) was performed (RT was substituted with water). The (−/+)RT reactions were incubated in a PCR cycler at 25 °C for 10 min, 50 °C for 30 min, and 85 °C for 5 min. Primers for quantitative PCR (qPCR) were designed by using the online primer design software Primer3 v.0.4.0^59^. Primer specificity (single product peak) was validated by melting profiles. Primer sequences, length, annealing temperature, amplicon length, and position on target are reported in Supplementary Table 3. cDNAs (50 ng) were amplified with SYBR^®^ Select Master Mix (Thermo Fisher Scientific) according to the manufacturer’s instruction with a final primer concentration of 400 nM. PCR reactions were run in 96-well optical plates sealed with optical adhesive cover on a qTOWER 2.0/2.2 instrument (Analytik Jena AG). The following thermal cycling conditions were used (SYBR Select Master Mix reference, standard cycling mode): 50 °C for 2 min, 95 °C for 2 min, 40 cycles of 95 °C for 15 s, and 60 °C for 1 min. Three synthetic RNA spike-ins were used for normalization. The 2(-delta delta Ct) method was applied to calculate the normalized target gene expression fold change, with the amplification efficiency (*E*) for each target gene, slope of standard curve (*S*), and mean threshold cycle (Ct)^60^.

### RNA spike-ins

Synthetic RNA spike-in controls are derived from selected RNAs of the ERCC RNA Spike-in Mix (Ambion) as described in ref. ^29^. Briefly, spike-ins (three unlabeled and three 4sU-labeled) are in vitro transcribed using the MEGAscript T7 kit (Ambion). In vitro transcription (IVT) of unlabeled spike-ins was performed following the manufacturer’s instruction. For IVT of 4sU-labeled spike-ins, 10% of UTP was substituted with 4-thio-UTP (Jena Bioscience). This is to ensure at least similar 4sU incorporation rates in the IVT as has been observed in human cell lines^61–63^. RNA spike-ins were purified with RNAClean XP beads (Beckman Coulter) following the manufacturer’s instructions. The final RNA spike-in pool contained equal amounts of all RNA spike-ins.

### TT-seq and RNA-seq

A detailed step-by-step protocol has been deposited in the protocols.io repository^64^. Two biological replicates of reactions including RNA spike-ins were performed essentially as described^29^. Experiments were performed using ∼3.6 × 10^7^ cells per biological replicate. K562 cells were kept at optimal growth conditions (Ctrl), or subjected to heat shock at 42 °C as described above for 15 min (HS15) or 30 min (HS30). Cells were labeled with 4-thiouridine (4sU) (Sigma-Aldrich) for the last 5 min. Raji B (CDK9^as^) cells were pretreated with solvent (0.05% v/v DMSO) or 5 µM 1-NA-PP1 (EMD Millipore) at 37 °C for 5 min. Afterward, Raji B (CDK9^as^) cells were kept at 37 °C (Ctrl) or shifted to 42 °C for additional 30 min (HS30), and 4sU-labeled for the last 5 min. An overview for all treatment conditions prior to 5 min of 4sU labeling is provided in Supplementary Table 1. Cells were harvested by centrifugation at 3000 × *g* for 2 min. Total RNA was isolated with QIAzol reagent (QIAGEN) according to the manufacturer’s instructions^65^ except for the addition of 150 ng RNA spike-in mix together with QIAzol. RNAs (150 µg) were sonicated to generate fragments of <10 kbp (total fragmented RNA) using AFAmicro tubes in a S220 Focused-ultrasonicator (Covaris Inc.). 4sU-labeled RNA was purified from 300 µg (K562) or 600 µg (Raji B CDK9^as^) of total fragmented RNA. Separation of labeled RNA was achieved with streptavidin beads (Miltenyi Biotec) as described^29,66^. Elution was performed using freshly prepared 100 mM DL-Dithiothreitol (Sigma-Aldrich). Prior to library preparation, 4sU-labeled RNA (TT-seq) and total fragmented RNA (RNA-seq) were purified using RNAClean XP beads (Beckman Coulter) and quantified by Qubit High Sensitivity RNA kit (Thermo Fisher Scientific). Input RNA was treated with HL-dsDNase (ArcticZymes) and used for strand-specific library preparation according to the Ovation Universal RNA-Seq System (NuGEN) with minor modifications. For “first strand primer premix preparation” only random primers were used. The user’s guide instructions were followed from “first strand synthesis using DNAse-treated RNA” to “adaptor cleavage.” The precise number of PCR amplification cycles was determined using ∼10% of the library and the KAPA HIFI Library Amp Real Time kit (Kapa Biosystems) following the manufacturer’s instructions. For the remaining 45 µL of library, the determined number of PCR cycles was used following the user guide’s instructions for amplification as described in “using qPCR to determine the number of PCR cycles.” The purified and preamplified fragments were analyzed on a Fragment Analyzer (Agilent) before clustering and sequencing on a NextSeq 550 (Illumina) in paired-end mode with 75 bp read length.

### TT-seq/RNA-seq preprocessing and normalization parameters

Paired-end 75 bp reads with additional six bases of barcodes were obtained for each of the samples (Supplementary Table 1). Reads were demultiplexed and mapped with STAR 2.3.0^67^ to the hg20/hg38 (GRCh38) genome assembly (Human Genome Reference Consortium). Samtools^68^ was used to quality filter SAM files, whereby alignments with MAPQ smaller than seven (−q 7) were skipped and only proper pairs (−f2) were selected. Further data processing was carried out using the R/Bioconductor environment. We used a spike-in (RNAs) normalization strategy essentially as described^29^ to allow observation of global shifts (sequencing depth) *σ*_*j*_, cross-contamination rate ${\it{\epsilon }}_j$ (proportion of unlabeled reads purified in the TT-seq samples), and antisense bias ratio *c*_*j*_ (ratio of spurious reads originating from the opposite strand introduced by the RT reaction). Read counts (*k*_*ij*_) for spike-ins were calculated using HTSeq^69^. Calculations for each parameter are described in the following in more detail.

### Antisense bias ratio *c*_*j*_

Antisense bias ratios were calculated for each sample *j* according to

1 $$c_j = \mathop {{{\mathrm{median}}}}\limits_i \left( {\frac{{k_{ij}^{{\mathrm{antisense}}}}}{{k_{ij}^{{\mathrm{sense}}}}}} \right)$$

with read counts *k*_*ij*_ for all available spike-ins *i* in sample *j*.

### Sequencing depth ***σ***_***j***_ and cross-contamination rate $\boldsymbol{\epsilon}$_***j***_

Sequencing depths were calculated for each sample *j* according to

2 $$\sigma _j = \mathop {{{\mathrm{median}}}}\limits_i \left( {\frac{{k_{ij}}}{{l_i}}} \right)$$

with read counts *k*_*ij*_ for all available spike-ins *i* in sample *j* for the RNA-seq samples and the labeled spike-ins *i* in sample *j* for the TT-seq samples. The cross-contamination rate ${\it{\epsilon }}_j$ was calculated for each sample *j* as

3 $${\it{\epsilon }}_j = \frac{{\mathop {{{\mathrm{median}}}}\limits_i \left( {\frac{{k_{ij}}}{{l_i}}} \right)}}{{\sigma _j}}$$

using the unlabeled spike-ins *i* for TT-seq samples. Note that ${\it{\epsilon }}_j$ is set to 1 for the RNA-seq samples.

### TUs based on the UCSC RefSeq (RefSeq-TUs)

For each annotated gene, TUs were defined as the union of all existing inherent transcript isoforms (UCSC RefSeq GRCh38).

### Isoform-independent exonic regions (constitutive exons)

Isoform-independent exonic regions were determined using a model for constitutive exons^70^ based on UCSC RefSeq annotation (GRCh38).

### GenoSTAN annotation of TUs

Annotation of different transcript classes was done as in ref. ^29^ with minor differences. In brief, genome-wide coverage was calculated from all TT-seq fragment midpoints in consecutive 200-bp bins throughout the genome. In order to create a comprehensive annotation independent of heat shock induced length differences, two replicate tracks were constructed by taking the maximum of each bin over the first and second replicates, respectively, regardless of treatment. A two-state hidden Markov model with a Poisson-log-normal emission distribution was learned in order to segment the genome into “transcribed” and “untranscribed” states. Consecutive “transcribed” states were joined, if its gaps were smaller than 200 bp, within a validated GENCODE mRNA or lincRNA (version 22) or showed uninterrupted coverage supported by all TT-seq samples. Subsequently, TU start and end sites were refined to nucleotide precision by finding borders of abrupt coverage increase or decrease between two consecutive segments in the four 200-bp bins located around the initially assigned start and stop sites via fitting a piecewise constant curve to the TT-seq coverage profiles for both replicates using the segmentation method from the R/Bioconductor package "tilingArray"^71^.

### GRO-cap TSS refinement of TUs (cTUs, K562)

For all TUs *i*, the GRO-cap refined tss* was determined as the closest nonzero GRO-cap signal^43^ in a window of 500 bp around the start of the TUs. Note that all TUs without an assigned GRO-cap site were not used. It was recently shown that upon 1 h of heat shock the TSS architecture remains mostly unchanged^44^. Thus, we assume changes of the TSS architecture upon 30 min of heat shock to be insignificant.

### Transcript sorting (K562 and Raji)

We sorted each gene (cTU for K562, TU for Raji) into one of the following seven classes: eRNA, sincRNA, asRNA, conRNA, uaRNA, lincRNA, and mRNA. First, (c)TUs reciprocally overlapping by at least 50% with a validated GENCODE mRNA or lincRNA (version 22) on the same strand were classified as mRNAs and lincRNAs. (c)TUs reciprocally overlapping by less than 50% with a validated GENCODE mRNA or lincRNA (version 22) on the same strand were not classified. Next, (c)TUs located on the opposite strand of either a mRNA or lincRNA were classified as asRNA if the TSS was located >1 kb downstream of the sense TSS on the opposite strand, as uaRNA if its TSS was located <1 kb upstream of the sense TSS, and as conRNA if its TSS was located <1 kb downstream of the TSS on the opposite strand. For K562, each of the remaining cTUs were classified as sincRNA. Every ncRNA (sincRNA, asRNA, conRNA, or uaRNA) was reclassified as eRNA if its TSS fell into a K562 enhancer state^42^. This resulted in 11324 non-ambiguously classified RNAs in K562 cells (825 eRNAs, 1581 sincRNAs, 564 asRNAs, 502 conRNAs, 1064 uaRNAs, 239 lincRNAs, and 6549 mRNAs). For Raji, each of the remaining TUs were classified into eRNA—if its TSS exhibited a high (>1) ratio H3K4me1/H3K4me3—or as sincRNA—if its TSS exhibited a low (<1) ratio of H3K4me1/H3K4me3. This resulted in 16452 non-ambiguously classified RNAs (3451 eRNAs, 3479 sincRNAs, 1398 asRNAs, 326 conRNAs, 565 uaRNAs, 243 lincRNAs, and 6990 mRNAs).

### Calculation of the number of transcribed bases

Of all sequenced fragments, only those were kept that exhibited a positive inner mate distance. The number of transcribed bases (tb_*j*_) for all samples was calculated as the sum of the coverage of evident (sequenced) fragment parts (read pairs only) for all fragments with an inner mate interval not entirely overlapping a Refseq annotated intron (UCSC RefSeq GRCh38, ~98.5% of all fragments) in addition to the sum of the coverage of nonevident fragment parts (entire fragment).

### TT-seq/RNA-seq processing with normalization parameters

The number of transcribed bases (tb_*j*_) or read counts (*k*_*j*_) for all features ((c)TUs, constitutive exons, or RefSeq-TUs) were normalized and corrected for antisense bias *c*_*j*_, sequencing depth *σ*_*j*_ (K562 and Raji), and cross-contamination rate ${\it{\epsilon }}_j$ (K562) as follows using the parameters calculated as described above. Note that cross-contamination rate estimates for TT-seq in Raji were very low and were thus not corrected for.

### Antisense bias correction

The real number of read counts or coverage *s*_*ij*_ for transcribed unit *i* in sample *j* was calculated as

4 $$s_{ij} = \frac{{S_{ij} - c_jA_{ij}}}{{1 - c_j^2}}$$

where *S*_*ij*_ and *A*_*ij*_ are the observed numbers of read counts or coverage on the sense and antisense strand.

### Sequencing depth and cross-contamination correction

The antisense bias corrected read counts or coverage *s*_*ij*_ of transcribed unit *i* in sample *j* was normalized for sequencing depth and cross-contamination as

5 $$t_{ij} = \frac{\frac{s_{ij}^{{\mathrm{TT}} - {\mathrm{seq}}}}{\sigma _j^{{\mathrm{TT}} - {\mathrm{seq}}}} - {\it{\epsilon }}_j \frac{s_{ij}^{{\mathrm{RNA}} - {\mathrm{seq}}}}{\sigma _j^{{\mathrm{RNA}} - {\mathrm{seq}}}}}{{1 - {\it{\epsilon }}_j}}$$

Note that *σ*_*j*_ was balanced between replicates via classical size factor normalization^72^ to gain statistical power in the differential expression analysis. Also note that, the cross-contamination rate ${\it{\epsilon }}_j$ was set to 0 in the formula above for TT-seq in Raji.

### Read counts per kilobase (RPK)

RPKs were calculated upon antisense bias corrected read counts (*k*_*j*_) falling into the region of a cTU, constitutive exon or RefSeq-TU divided by its length in kilobases.

### Number of transcribed bases per base (coverage)

Coverages were calculated upon antisense bias corrected number of transcribed bases (tb_*j*_) falling into the region of a cTU divided by its length in bases. Based on the antisense bias corrected coverages a subgroup of expressed cTUs was defined to comprise all cTUs with a coverage of five or higher in one of two summarized replicates of TT-seq (HS15, HS30, or control).

### mNET-seq

Two biological replicates of reactions including Empigen BB detergent treatment during IP were performed essentially as described^28,45,73^. See Supplementary Table 1 for an overview of experimental conditions and biological replicates. Briefly, cells were kept at optimal growth conditions (Ctrl) or subjected to heat shock at 42 °C as described above for 15 min (HS15) or 30 min (HS30). Experiments were performed using 1.0 × 10^8^ K562 cells per biological replicate. Per reaction, biochemical fractionations of K562 cells were performed as described^74^. All buffers were freshly prepared and complemented with Protease Inhibitor Cocktail (Sigma-Aldrich) and PhosSTOP (Roche). Isolated chromatin was digested with 50 U micrococcal nuclease (MNase) (NEB) at 37 °C and 1400 r.p.m. in a ThermoMixer (Eppendorf) for 2 min. To inactivate MNase, EGTA (Bioworld) was added to a final concentration of 25 mM. Digested chromatin was collected by centrifugation at 4 °C and 16,000 × *g* for 5 min. The supernatant was diluted eightfold with IP buffer containing 50 mM Tris-HCl pH 7.4, 150 mM NaCl, 0.05% v/v NP-40, and 1% v/v Empigen BB (~30% active substance, Sigma-Aldrich). For IP, 30 µg of antibody targeting total Pol II, i.e., hRPB1 (phosphorylated and unphosphorylated) CTD (MABI0601; BIOZOL), was conjugated to Dynabeads M-280 Sheep Anti-Mouse IgG (Thermo Fisher Scientific) (Supplementary Table 4). The IP was performed on a rotating wheel at 4 °C for 1 h. The beads were washed six times with IP buffer (50 mM Tris-HCl pH 7.5, 150 mM NaCl, 0.05% NP-40, and 1% Empigen BB) and once with 500 µL of PNKT buffer containing 1 × T4 polynucleotide kinase (PNK) buffer (NEB) and 0.1% v/v Tween-20 (Sigma-Aldrich). Beads were incubated in 100µL of PNK reaction mix containing 1 × PNK buffer, 0.1% v/v Tween-20, 1 mM ATP, and T4 PNK 3′ phosphatase minus (NEB) at 37 °C for 10 min. Beads were washed once with IP buffer. RNA was extracted with TRIzol reagent (Thermo Fisher Scientific), precipitated in ethanol, and resolved on a 6% denaturing PAGE containing 7 M urea for size purification^75^. Bromophenol blue and xylene cyanol were used as tracking dyes. To select fragments of 25–110 nt, the gel fragment was cut between the two tracking dyes, eluted from the gel by the crush-and-soak method using elution buffer (1 M NaOAc, 1 mM EDTA), and precipitated in ethanol. RNA libraries were prepared according to the TruSeq Small RNA Library Kit (Illumina) and as described in ref. ^73^. For final size selection of the amplified library, 4% E-Gel™ High-ReSolution Agarose Gels (Invitrogen) were used. The size-selected fragments were analyzed on a Fragment Analyzer (Agilent) before clustering and sequencing on a NEXTseq 550 (Illumina) in paired-end mode with 75bp read length.

### mNET-seq data preprocessing

Paired-end 75 bp reads with additional six bases of barcodes were obtained for each of the samples (Supplementary Table 1). Reads were demultiplexed, trimmed for adapter content with cutadapt^76^ (-O 12 -m 25 -a TGGAATTCTCGG -A GATCGTCGGACT), and mapped with STAR 2.3.0^67^ to the hg20/hg38 (GRCh38) genome assembly (Human Genome Reference Consortium). Samtools^68^ was used to quality filter SAM files, whereby alignments with MAPQ smaller than 7 (−q 7) were skipped and only proper pairs (−f2) were selected. Further data processing was carried out using the R/Bioconductor environment. Antisense bias (ratio of spurious reads originating from the opposite strand introduced by the RT reactions) was determined using positions in regions without antisense annotation with a coverage of at least 100 according to Refseq-TUs (UCSC RefSeq GRCh38). Coverage tracks for further analysis were restricted to the last nucleotide incorporated by the Pol in the aligned mNET-seq reads.

### mNET-seq data normalization

We first identified a subgroup of RefSeq-TUs with unchanged behavior over the response to heat shock in the spike-ins normalized TT-seq data via k-means clustering. On the resulting 3416 RefSeq-TUs *i*, size factors for each sample *j* were determined as

6 $$\sigma _j = \mathop {{{\mathrm{median}}}}\limits_i \left( {\frac{{p_{ij}}}{{\left( {\mathop {\prod }\nolimits_{v = 1}^m p_{ij}} \right)^{\frac{1}{m}}}}} \right)$$

where *m* denotes the total number of antisense corrected mNET-seq samples (*p*_*ij*_). This formula has been adapted^72^ and was used to correct for library size and sequencing depth variations.

### Detection of pause sites

For all expressed (c)TUs or RefSeq-TUs (exceeding 10 kbp in length with one unique TSS given all RefSeq annotated isoforms (UCSC RefSeq GRCh38)) *i* the pause site n* was calculated for all bases *m* in a window of 350 bp downstream of the GRO-cap refined TSS and in a window from the TSS to the end of the first exon (excluding the last five bases) for RefSeq-TUs via maximizing the function

7 $$\rho _i = \mathop {{\max }}\limits_m p_{im}$$

where *ρ*_*i*_ needed to exceed five times the median of the signal strength *p*_*im*_ for all nonnegative antisense bias corrected mNET-seq coverage values. In order to maximize the chances of finding the most likely pause site, two replicate tracks were constructed by taking the maximum of each nucleotide over the first and second replicates, respectively, regardless of treatment. We defined the pause site at the active site based on structural information^15^. The RNA-DNA hybrid within the paused Pol is in the tilted state that hinders nucleotide addition at the active site. Thus, the subsequent nucleotide is not added yet. We defined the pause site to be the position in line with the “posttranslocated” RNA rather than with the ‘pretranslocated'' DNA. In conclusion, the pause site was calculated as $n^ \ast = m^ \ast + 1$, where m* is the argument that maximizes *ρ*_*i*_. For K562, this resulted in 10363 expressed, non-ambiguously classified RNAs (604 eRNA, 471 asRNA, 1314 sincRNA, 965 uaRNA, 445 conRNA, 209 lincRNA, and 6355 mRNA). For Raji, this resulted in 8145 expressed, non-ambiguously classified RNAs (501 eRNA, 318 asRNA, 500 sincRNA, 461 uaRNA, 253 conRNA, 145 lincRNA, and 5967 mRNA).

### Molecular weight conversions

The known sequence and mixture of the utilized spike-ins allow to calculate a conversion factor to RNA amount per cell [cell^−1^] given their molecular weight assuming perfect RNA extraction. The number of spike-in molecules per cell *N* [cell^−1^] was calculated as

8 $$N = \frac{m}{{Mn}}N_A$$

with the mass *m* 25 × 10^−9^ [g] per spike-in, the number of cells *n* (3.8 × 10^7^ for K562, 3.4 × 10^7^ for Raji), the Avogadro constant *N*_*A*_ 6.02214085774 × 10^23^ [mol^−1^] and molar mass (molecular weight) of the spike-ins *M* [g mol^−1^] calculated as

9 $$M = \,\, A_n \times 329.2 + \left( {1 - \tau } \right) \times U_n \times 306.2 + C_n \times 305.2 + G_n \times 345.2 \\ \!\! +\, {\mathrm{\tau }} \times U_n \times 322.26 + 159 + {\mathrm{\tau }} \times U_n \times 322.26 + 159$$

where *A*_*n*_, *U*_*n*_, *C*_*n*_, and *G*_*n*_ are the number of each respective nucleotide within each spike-in polynucleotide. τ is set to 0.1 in case of a labeled spike-in and 0 otherwise (${\mathrm{\tau }} \cdot U_n$ corresponds to the number of 4sU nucleotides, 4sU_*n*_). The addition of 159 to the molecular weight considers the molecular weight of a 5′ triphosphate. Provided the above the conversion factor to RNA amount per cell *κ* [cell^−1^] can be calculated as

10 $$\kappa = {\mathrm{mean}}\left( {\mathrm{median}_{i}} \left({\frac{{tb_i}}{{L_i \times N}}}\right) \right)$$

for all labeled spike-in species *i* with length *L*_*i*_. Note that imperfect RNA extraction efficiency would lead to an underestimation of cellular labeled RNA in comparison to the number of added spike-ins and thus to an underestimation of initiation frequencies. In case of a strong underestimation, however, the real initiation frequencies would lie above the pause-initiation limit, which is theoretically impossible. Thus, we assume this effect to be insignificant.

### Estimation of productive initiation frequency *I*

The antisense bias corrected number of transcribed bases $tb_i^{condition}$ was calculated on all (c)TUs for treatment (42 °C, or CDK9 inhibition), and control (37 °C, or solvent) or expressed RefSeq-TUs (exceeding 10 kbp in length with one unique TSS given all RefSeq annotated isoforms (UCSC RefSeq GRCh38)). For each (c)TU or RefSeq-TU *i* the productive initiation frequency *I*_*i*_ [cell^−1^ min^−1^], which corresponds to the pause release rate, was calculated as

11 $$I_i = \frac{1}{\kappa } \times \frac{{tb_i^{{\mathrm{condition}}}}}{{t \times L_i}}$$

with labeling duration *t* = 5 [min] and length *L*_*i*_. Note that for RefSeq-TUs, $tb_i^{condition}$ and *L*_*i*_ were restricted to regions of nonfirst constitutive exons (exonic bases common to all isoforms) located in the first 25 kbp. Given the 15- and 30-min heat shock treatment, we expect only the first 35 kbp to be significantly affected by changes in initiation frequency assuming an average elongation velocity of 2.4 kbp min^−1^ ^28^. In addition, changes in splicing rate upon heat shock treatment^77,78^ should not influence constitutive exonic regions. This is to ensure, not to be biased by alternative splicing.

### Estimation of pause duration *d*

For all cTUs for 15 and 30 min heat shock treated (42 °C), and control (37 °C) or expressed RefSeq-TUs (exceeding 10 kbp in length with one unique TSS given all RefSeq annotated isoforms (UCSC RefSeq GRCh38)) the pause duration *d*_*i*_ [min] was calculated as the residing time of the Pol in a window +/−100 bases *m* around the pause site (see above) as

12 $$d_i = \frac{{\sum \nolimits_{ + / - 100} p_{im}}}{{I_i}} \times {\mathrm{median}_{i}} \left( {\frac{{v_i}}{{\frac{{I_iv_i\left( {t^ \ast - t} \right)}}{{\sum\nolimits_{{\mathrm{response}\,\mathrm{window}}} p_{im}}}}}} \right)$$

with pause release rate *I*_*i*_ and the number of polymerases *p*_*im*_ (antisense bias corrected mNET-seq coverage values) in a window +/−100 bases around the pause site. For pause sites less than 100 bp downstream of the TSS the first 200 bp of the cTU were considered. Note that the right part of the formula is restricted to mNET-seq instances above the 50% quantile for robustness and adjusts *d*_*i*_ to an absolute scale by comparing the heat shock response derived elongation velocities *ν*_*i*_ with those derived from combining mNET-seq and TT-seq data in the response window $\left[ {200,v_i\left( {t^ \ast - t} \right)} \right]$. The productive initiation frequency represents the “true” initiation frequency if the fraction of Pol II terminating within the pause window is insignificant (unknown fraction of early termination). Note that the pause duration *d* obtained in this way reflects the effective pause between two initiation events that successfully lead to productive elongation of a transcript and thus the relevant transcriptional outcome. Thus, our model is independent of the exact mechanism at the promoter-proximal pause site, may it be pausing or premature termination.

### Calculation of response ratios (for calibration of *d*)

For each condition *j* (control or heat shock 15 min) the antisense bias corrected number of transcribed bases $tb_i^j$ was calculated on all expressed RefSeq-TUs *i* (exceeding 35 kbp in length with one unique TSS given all Refseq annotated isoforms (UCSC RefSeq GRCh38)). Response ratios were calculated for a window from the TSS to 10 kbp downstream (excluding the first 200 bp) for each RefSeq-TU *i* as

13 $$r_i = 1 - \frac{{\mathrm{tb}_{i_{\left[ {0.2,\,10{\mathrm{kbp}}} \right]}}^{\mathrm{heat}\,\mathrm{shock}}}}{{\mathrm{tb}_{i_{\left[ {0.2,\,10{\mathrm{kbp}}} \right]}}^{\mathrm{control}}}}$$

where negative values were set to 0 and values above 1 were set to 1.

### Estimation of elongation velocity (for calibration of *d*)

For each condition *j* (control or heat shock 15 min) the antisense bias corrected number of transcribed bases $tb_i^j$ was calculated on all expressed RefSeq-TUs *i* (exceeding 35 kbp in length with one unique TSS given all Refseq annotated isoforms (UCSC RefSeq GRCh38)), excluding the first 200 bp. All TUs were truncated by 5 kbp in length from the 3′ end prior to calculation to avoid influence of some alterations in signal around the pA site after heat shock. For each TU *i* with $r_i > 0.1$ the elongation velocity *v*_*i*_ [kbp min^−1^] was calculated as

14 $$v_i = \frac{{{\mathrm{tb}}_i^{{\mathrm{control}}} - {\mathrm{tb}}_i^{{\mathrm{heat}\,\mathrm{shock}}}}}{{{\mathrm{tb}}_i^{{\mathrm{control}}} \times \frac{{r_i}}{{L_i}}\left( {t^ \ast - t} \right)}}$$

with heat shock treatment duration t^*^ = 15 [min] and labeling duration *t* = 5 [min].

### Pause-initiation limit

The previously derived inequality from^27^

15 $$\frac{v}{I} \ge 50\,\mathrm{[bp]}$$

states that new initiation events into productive elongation are limited by the velocity of the polymerase in the promoter-proximal region and that steric hindrance occurs at distance of less than 50 bp between the active sites of the initiating Pol II and the paused Pol II^28^. Given the calculations of pause duration *d* and (productive) initiation frequency *I* above, we can reformulate this inequality to

16 $$\frac{{200\,\mathrm{{[bp]}}}}{{d \times I}} \ge 50\,\mathrm{[bp]}$$

with 200 [bp] being the above defined pause window.

### Prediction of RNA secondary structure (MFE)

The gene-wise mean minimum free energy (MFE) for a window of [−15,−65] bp upstream of the pause site was calculated from subsequent MFE estimates of 13-bp RNA fragments tiling the respective area using RNAfold from the ViennaRNA package^79^.

### HSF1 driven enhancers and promoters

cTUs were classified as HSF1 driven, if a HSF1 binding site was found in a window of 1500 bp upstream to 500 bp downstream of the TSS based on HSF1 binding events (peak calls) that were determined in heat shock conditions of cycling K562 cells (Vihervaara et al.^35^, data availability: GSE43579).

### Reporting summary

Further information on research design is available in the Nature Research Reporting Summary linked to this article.

## Supplementary information

Supplementary information

Peer Review

Reporting summary

Source data

## Data Availability

The data that support the findings of this study are available from the corresponding author upon reasonable request. The complete set of mNET-seq, TT-seq, and RNA-seq sequencing data and processed files generated for this study was deposited in the GEO database (http://www.ncbi.nlm.nih.gov/geo) under accession code GSE123980. A list of all publicly available datasets and the corresponding accession codes used in this study is provided in Supplementary Table 5. The source data underlying Supplementary Fig. 3 (RT-qPCR, cell counts) are provided as a Source Data file.

## Code availability

Computational analyses have been performed using R/Bioconductor. Custom scripts can be made available upon request. A detailed description of the bioinformatic workflow used to analyze TT-seq/RNA-seq data has been deposited in the bioRxiv preprint server^80^.
