# Supplementary material for: The pause-initiation limit restricts transcription activation in human cells
Source: Nat Commun. 2019 Aug 9;10:3603. doi: 10.1038/s41467-019-11536-8 (PMC6689055; doi:10.1038/s41467-019-11536-8)
Supplement: Supplementary file 1 — Supplementary information [file 41467_2019_11536_MOESM1_ESM.pdf]

Supplementary information for 'The pause-initiation limit restricts transcription activation in human cells'

Supplementary note 1 .... p. 1
Supplementary figures 1-10 .... p. 2-14
Supplementary tables 1-5 .... p. 15-22
References .... p. 23

Supplementary note

Supplementary Note 1. **Occupancy profiling alone does not reveal kinetics.**

To test whether Pol II pausing behavior can be inferred from Pol II occupancy changes alone, we investigated our mNET-seq data after 15 and 30 min heat shock response (Supplementary Fig. 6 e). After 15 min of heat shock, the average mNET-seq signal for upregulated genes increased in the pause window and in the gene body, and this does not allow for conclusions. After 30 min of heat shock, the signal had increased further in the gene body but not in the pause window, an observation that could have been correctly interpreted as a decrease in *d* and an increase in *I* (Supplementary Fig. 6 e). For downregulated genes after 15 min of heat shock, the signal in the pause window remained unchanged, whereas the signal in the gene body decreased, again not allowing for definitive conclusions with respect to the duration of pausing, because the same profile change is expected by a decrease in productive initiation frequency. After 30 min of heat shock, the signal in the pause window increased, whereas the signal in the gene body decreased further, and this could be interpreted as an increase in *d* and a decrease in *I* (Supplementary Fig. 6 e). Assuming unchanged elongation velocity in the gene body upon heat shock[1], these Pol II occupancy changes are generally consistent with reduced and increased pause durations for up- and down-regulated genes, respectively, although the interpretation of mNET-seq data at single time points alone would not always have led to the correct conclusions. Taken together, the observed changes in Pol II pausing behavior may in favorable cases be inferred from an analysis of occupancy changes alone, but this is not always conclusive and cannot provide kinetics.

Supplementary figures

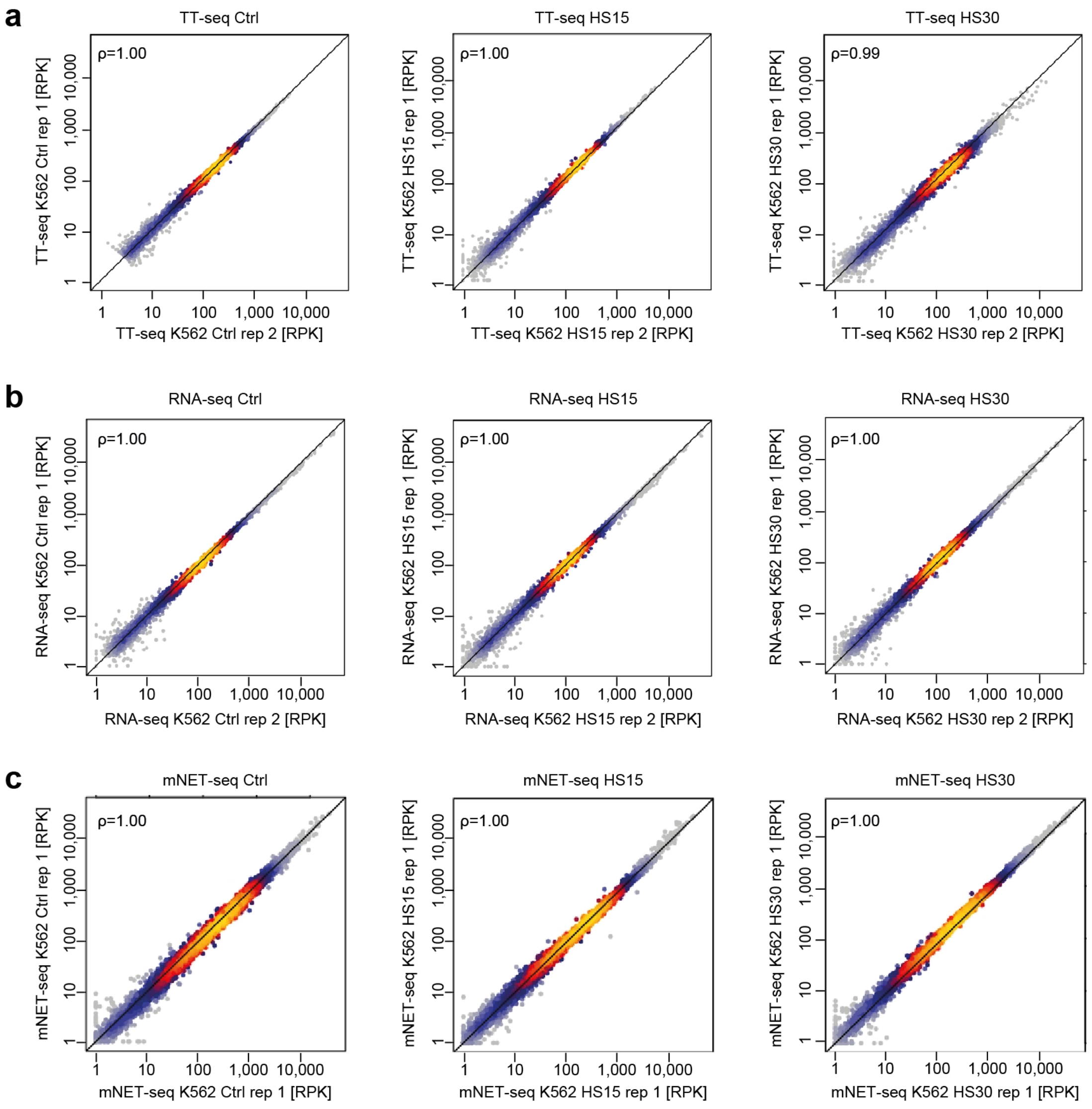

Supplementary Figure 1. **Correlation between biological replicates of TT-seq, RNA-seq, and mNET-seq in K562 cells for control and upon heat shock conditions.**

(a-c) TT-seq (a), RNA-seq (b), and mNET-seq (c) experiments were performed from two independent biological replicates for control (Ctrl), or heat shock (42 °C) conditions for 15 min (HS15) and 30 min (HS30) (Methods, Supplementary Table 1-2). Scatter plots with color-coded density of reads for 12,315 RefSeq GRCh38 annotated protein-coding genes. Both axes depict reads per kilobase (RPK). The color scale corresponds to the density of points. Correlation of replicates is calculated as Spearman's rank correlation coefficient (rho) rounded to second decimal place (shown in each plot).

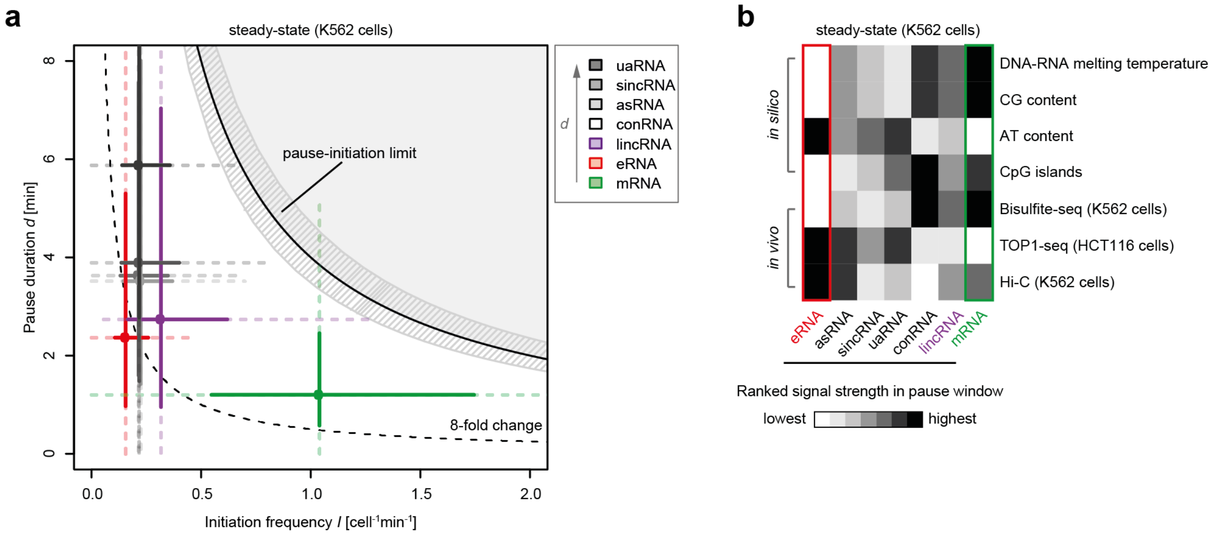

Supplementary Figure 2. **Transcription kinetics of different transcript classes in steady-state.**

(a) Plot shows the median productive initiation frequency I [cell^{-1}min^{-1}] depicted against the median pause duration d [min] for all transcript classes (circles). The two solid perpendicular lines define the inter-quartile range, the dotted whiskers represent 1.5 times the inter-quartile range of the respective estimate for the entire transcript class. The grey shaded area depicts impossible combinations of I and d[2, 3]. Striped area shows confidence intervals of the pause-initiation limit. The dotted line defines an 8-fold possible fold change until a gene would be restricted by the pause-initiation limit. (b) Chromatin features in the pause window (Methods) of different transcript classes. Data are ranked by each row across different transcript types to better highlight the contrast of individual features. Published data sets are listed in Supplementary Table 5.

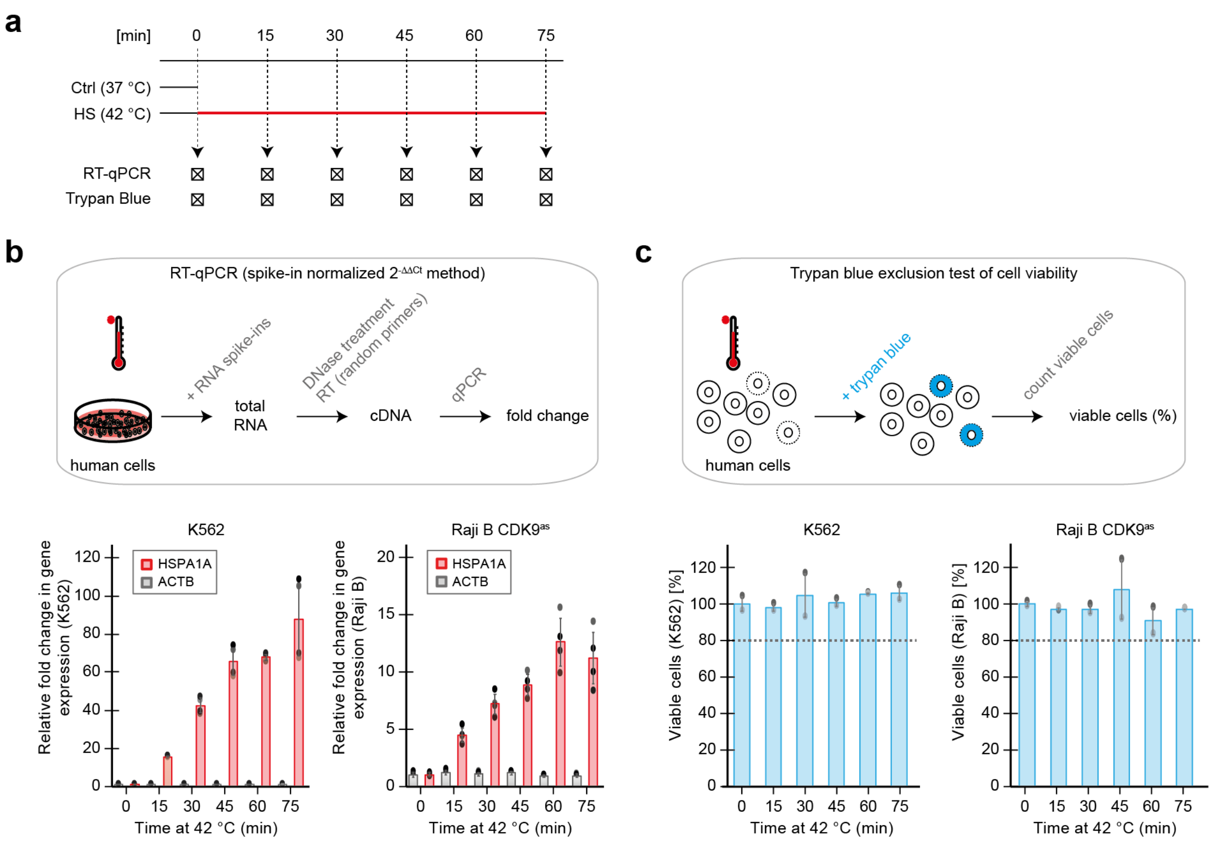

Supplementary Figure 3. **Differential expression of hsp70 (HSPA1A) and viability of human hematopoietic cells upon heat shock time-course.**

(a) Human K562 or Raji B (CDK9^{as}) cells were subjected to a heat shock (42 °C) time-course of 0 to 75 min. Every 15 min, total RNA was isolated and analyzed by RT-qPCR. Cell viability was tested by trypan blue exclusion test (Methods). (b) Top: experimental set-up of RT-qPCR of heat shock time-course. Bottom: relative fold change in gene expression of β-actin (ACTB, in grey), or hsp70 (HSPA1A, in red) in K562 (left bar plot) or Raji B (CDK9^{as}) (right bar plot) cells (spike-ins normalized). Error bars represent the standard deviation. Differential expression observed for HSPA1A in K562 and Raji B (CDK9^{as}) cells agrees very well with other studies[4]. (c) Top: experimental set-up of trypan blue exclusion assay. Bottom: viable cells [%] for K562 (left bar plot) or Raji B (CDK9^{as}) (right bar plot) upon heat shock time-course. Source data are provided as a Source Data file 1.

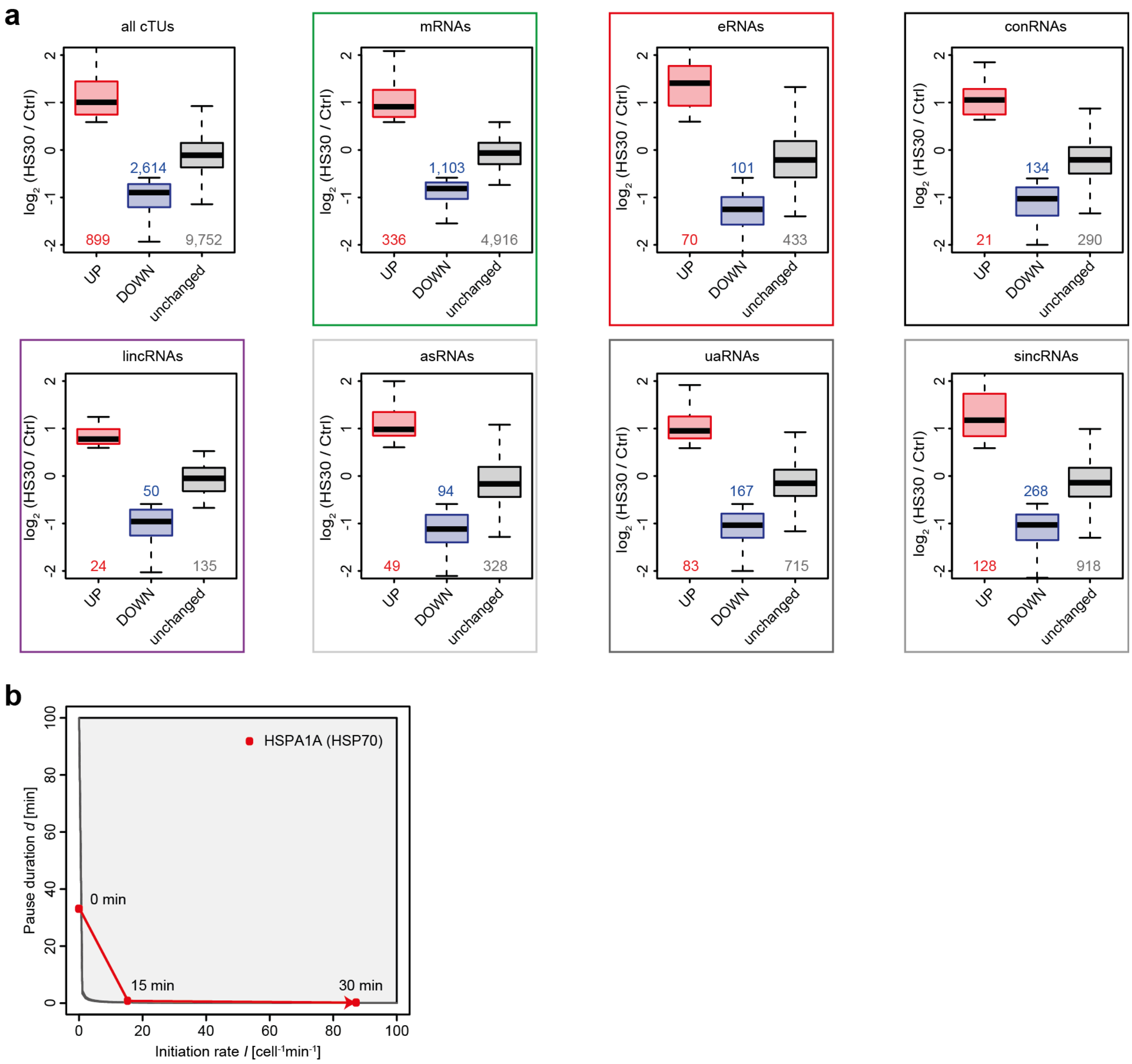

Supplementary Figure 4. **Differential expression analysis of transcript classes and kinetics of hsp70 (HSPA1A) in human K562 cells upon heat shock.**

(a) Transcript classes as annotated in this study (Fig. 1; Methods). Boxplots show differential expression (DE) analysis of upregulated (light red), downregulated (dark blue), and unchanged genes (grey) in human K562 cells upon 30 min of heat shock (42 °C). Heat shock samples (HS30) were compared to respective control (Ctrl). Spike-in normalized TT-seq data were analyzed for DE of all genes (cTUs), mRNAs (green frame), eRNAs (light red frame), conRNAs (black frame), lincRNAs (purple frame), asRNAs, uaRNAs, sincRNAs (frame in different shades of grey). A minimum fold change of 1.5 in all conditions and a maximum adjusted p-value of 0.1 was set for calling a significant expression change. (b) Plot shows the median productive initiation frequency I [$cell^{-1}min^{-1}$] depicted against the median pause duration d [min] for hsp70 (HSPA1A) (red) for 0, 15, and 30 min at 42 °C (in red). The grey shaded area depicts impossible combinations of I and d[2, 3].

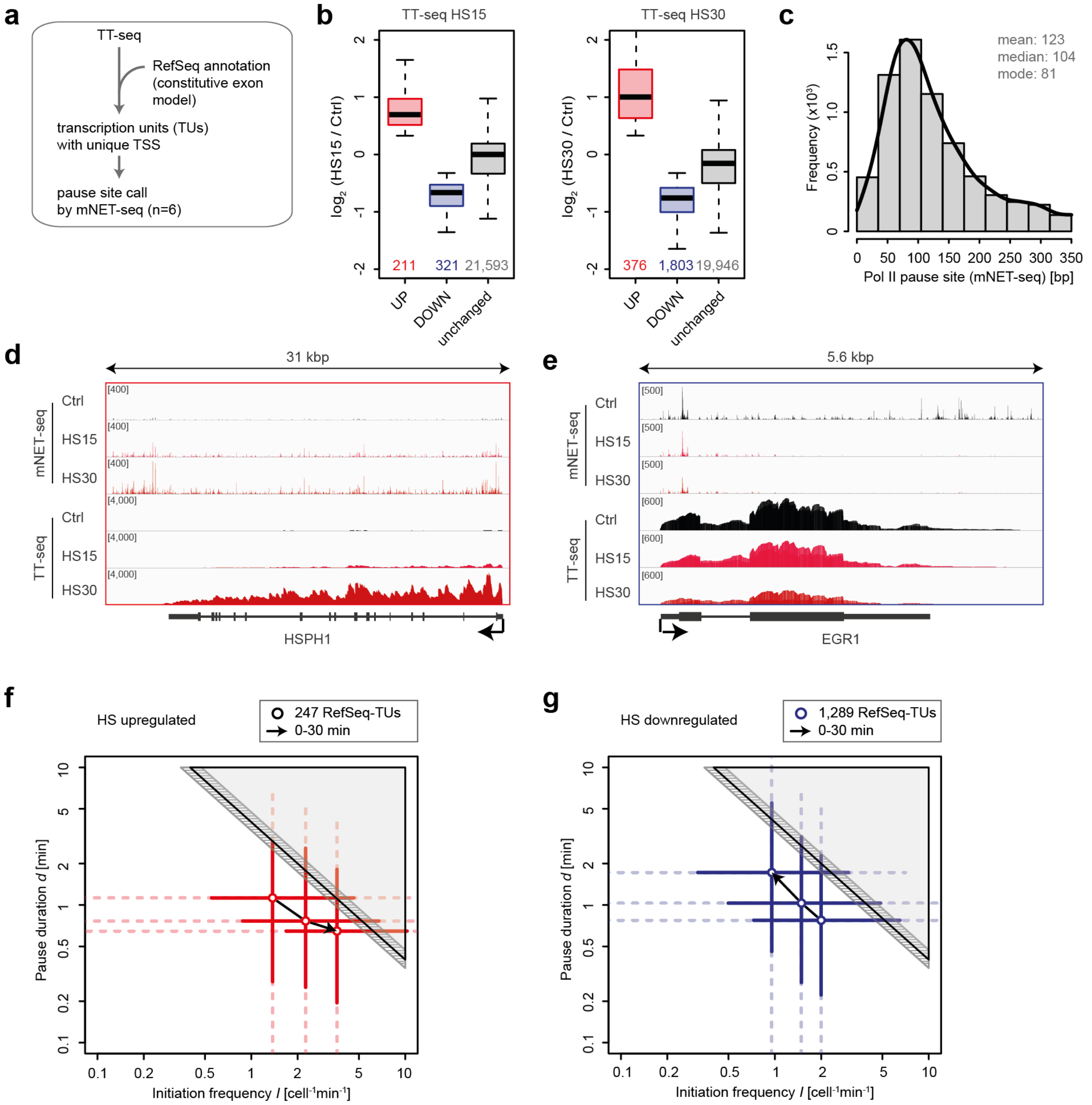

Supplementary Figure 5. **Conservative analysis of pause-initiation trajectories on constitutive exons agrees with analysis on new annotation.**

(a) Diagram illustrating the main selection steps of constitutive exons in all RefSeq GRCh38 annotated isoforms (Methods). (b) Differential expression (DE) analysis of upregulated (light red), downregulated (dark blue), and unchanged TUs (grey) in human K562 cells upon 15 min (left box plot) or 30 min (right box plot). Heat shock samples (HS15, or HS30) were compared to respective control (Ctrl). Spike-in normalized TT-seq data were analyzed for DE of constitutive exons in all RefSeq GRCh38 annotated isoforms[5]. A minimum fold change of 1.25 as cutoff and a maximum adjusted p-value of 0.001 was set for calling a significant expression change. Black bars represent medians, boxes mark upper and lower quartiles, whiskers represent 1.5 times the inter-quartile range. (c) Distribution of pause site distance from the TSS for 7,406 investigated genes measured by mNET-seq depicted as a histogram with respect to TSS of RefSeq annotated genes (RefSeq-TUs) (mean 123 [bp], median 104 [bp], mode 81

[bp]). Note that this in contrast to GRO-cap refined TSSs of our new K562 annotation for which called pause sites were distributed around a maximum located ~50 bp downstream of the TSS (Fig. 1 c). (d-e) Representative genome browser view of mNET-seq and TT-seq data at the HSPH1 gene locus on chromosome 13 (minus strand) (d), or at the EGR1 gene locus on chromosome 5 (plus strand) (e). Visualized with the Integrative Genomics Viewer (IGV, version 2.4.10; human hg38)[6]. Shown is the strand-specific Pol II occupancy with single nucleotide resolution (mNET-seq, top panels), and the number of transcribed bases (TT-seq, bottom panels). Two biological replicates are merged. (f-g) Median of pause-initiation trajectories upon heat shock time-course of 247 significantly upregulated (red circles, f) or 1,289 downregulated genes (dark blue circles, g) in log scale. The two solid perpendicular lines define the inter-quartile range, the dotted whiskers represent 1.5 times the inter-quartile range of the respective estimate for the entire transcript class. The grey shaded area depicts impossible combinations of I and d[2, 3]. Striped areas show confidence intervals of the pause-initiation limit.

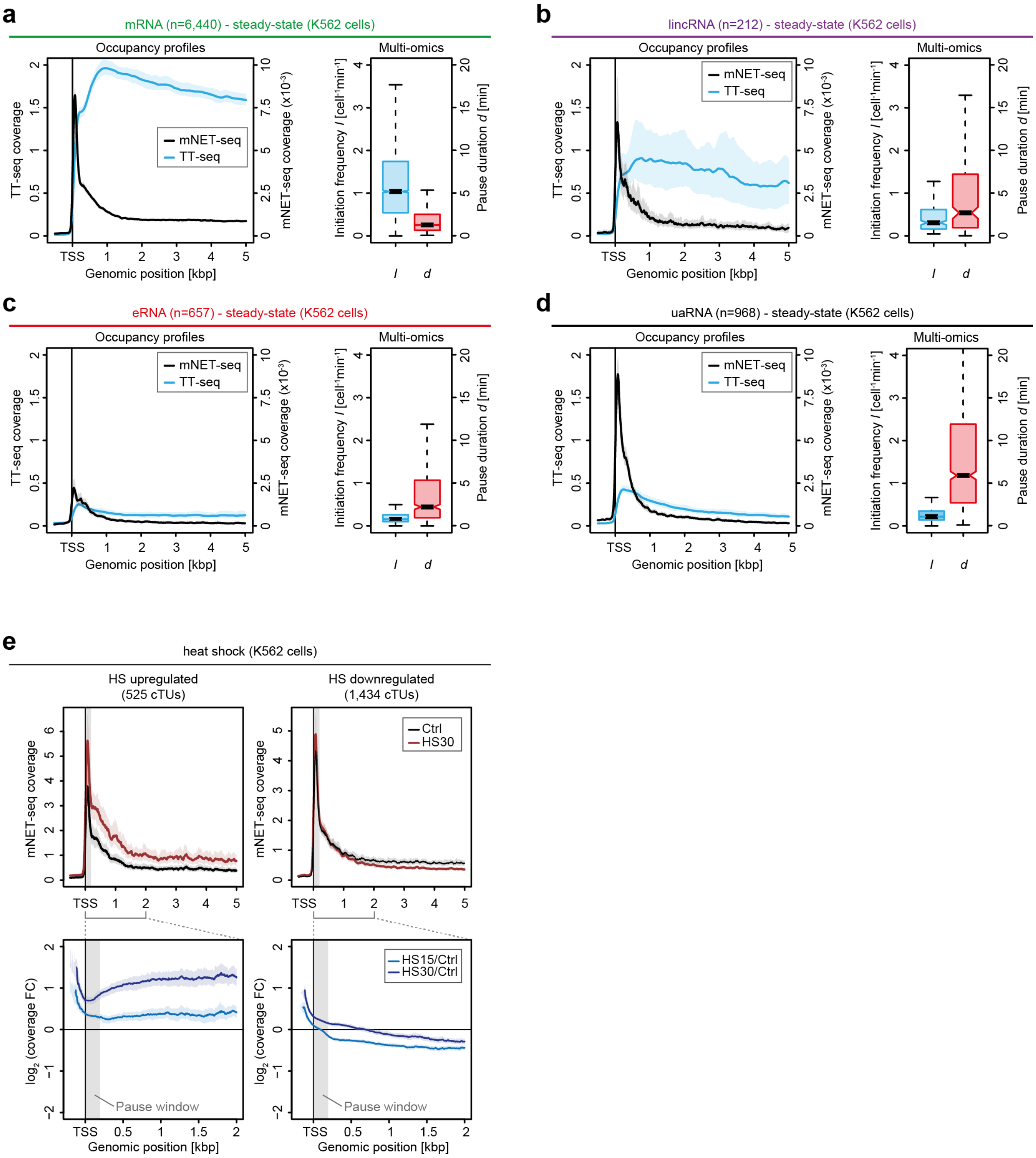

Supplementary Figure 6. **Multi-omics, but not occupancy profiling alone, can reveal transcription kinetics.**

(a-d) For comparison among different transcript classes, TT-seq coverage (left y-axis) and mNET-seq coverage (right y-axis) show the same range for all metagene profiles. Coverage per cell is shown for two biological replicates of steady-state K562 cells. (a-b) mRNAs (6,440, green) and lincRNAs (212, purple) show similar total Pol II peak heights in mNET-seq (with Empigen BB). However, the TT-seq signal is significantly lower for lincRNAs indicating less productive initiation events compared to mRNAs. (c-d) eRNAs (657, red) and uaRNAs (968, black) show a similar height of the TT-seq signal. However, Pol II occupancy strongly varies these transcript classes. As a consequence, our multi-omics approach shows that pause durations are longer for uaRNAs than for eRNAs. (e) Top: mNET-seq coverage aligned at the

TSS for 525 significantly upregulated (left), and 1,434 downregulated genes (right) with a minimum length of 5 kbp upon 30 min of heat shock (HS). Bottom: close-up showing coverage fold change upon 15 min (light blue) and 30 min (dark blue) of heat shock for genes as in top panel. Shaded areas show confidence intervals. The pause window is highlighted as grey shaded area (Methods).

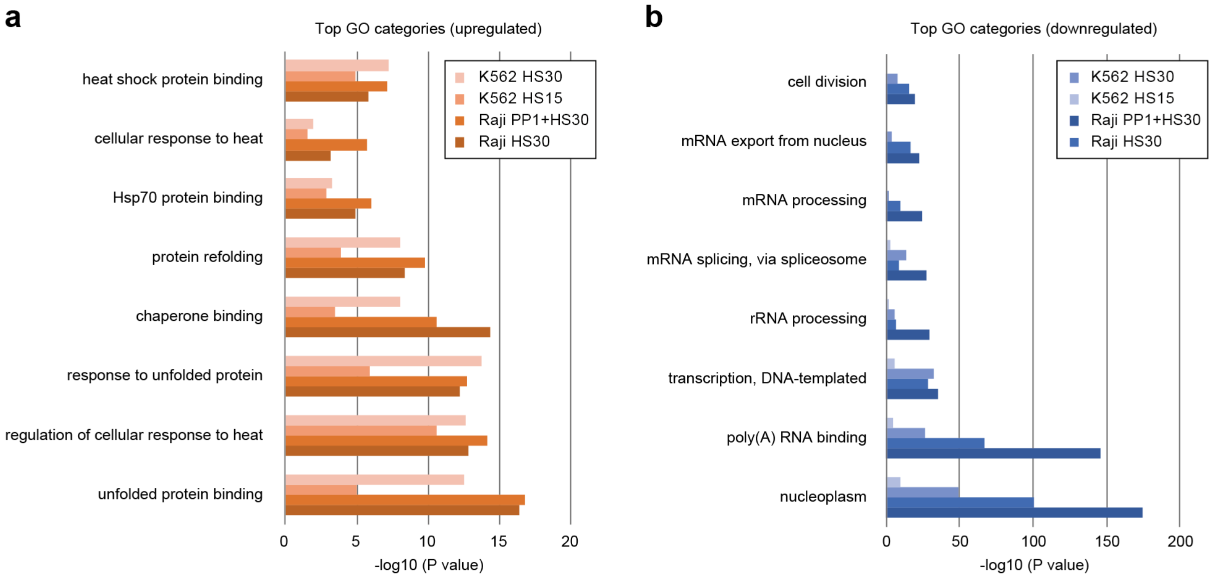

Supplementary Figure 7. **Gene ontology analysis of differentially expressed RefSeq-TUs in human hematopoietic cells exposed to heat shock.**

(a-b) Gene Ontology (GO) analysis[7] of significantly overrepresented categories linked to upregulated (a) or downregulated (b) categories in heat shock for human K562 or Raji B (CDK9[as]) cells. For K562 cells, heat shock samples (HS15, or HS30) were compared to respective control (Ctrl). For Raji B (CDK9[as]) samples, solvent heat shock sample (HS30) was compared to solvent control, while CDK9 inhibited heat shock sample (PP1+HS30) was compared to CDK9 inhibited control. Two biological replicates were averaged per condition and time-point.

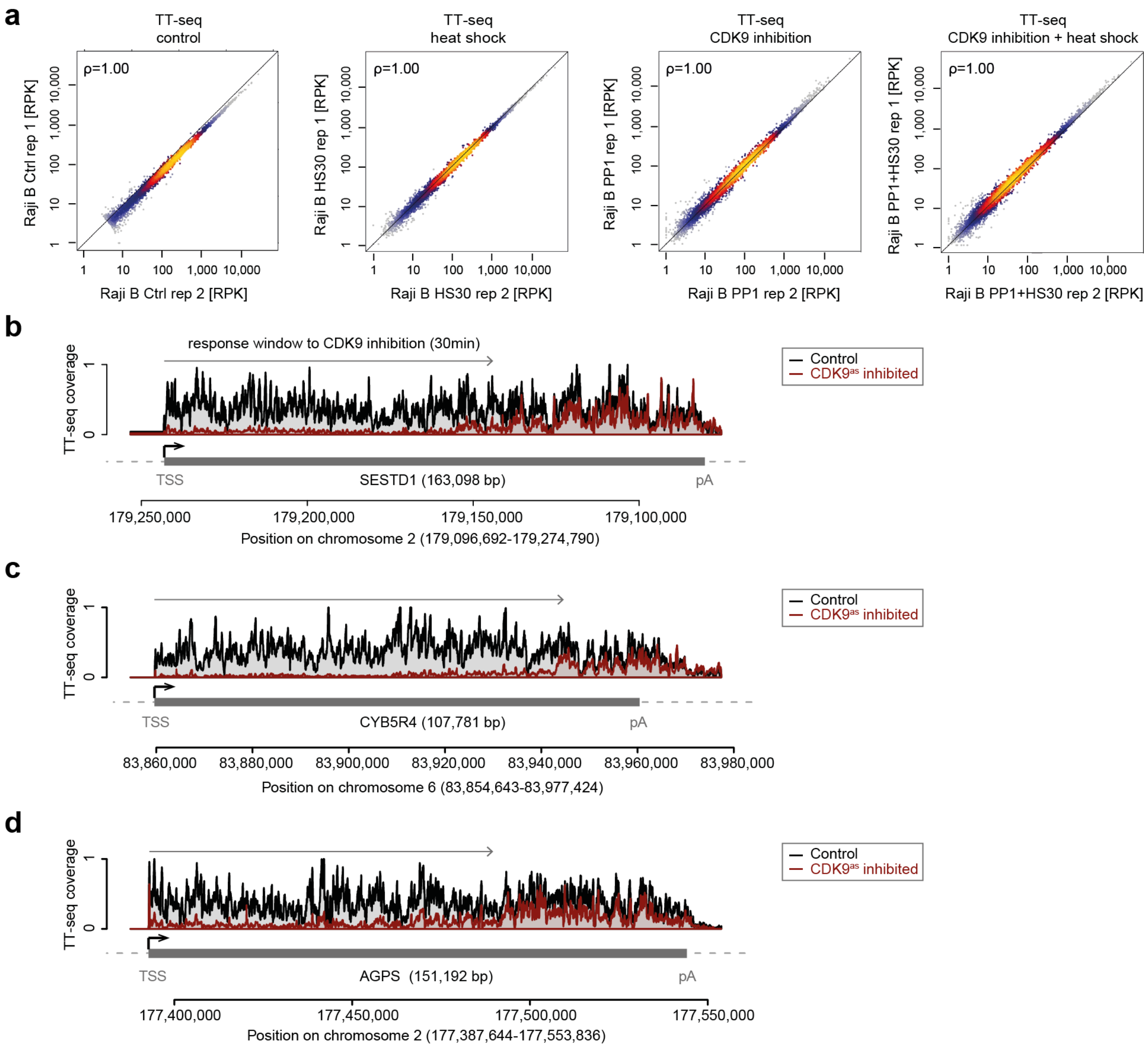

Supplementary Figure 8. **Analog-sensitive CDK9 inhibition in human Raji B (CDK9^{as}) cells.** (a) TT-seq experiments were performed from two independent biological replicates for control (Ctrl), or heat shock conditions for 30 min (HS30) with solvent DMSO or CDK9 inhibition (PP1) (Methods; Supplementary Table 1-2). Scatter plots show the density of reads of 10,294 RefSeq annotated protein-coding genes. Both axes depict reads per kilobase (RPK). The color scale corresponds to the density of points. Correlation of replicates is calculated as Spearman's rank correlation coefficient (rho) rounded to second decimal place (shown in each plot). (b-d) Based on the assumption that the inhibitor is evenly distributed across cells and within cells, the portion of CDK9^{as} molecules that has been fully inhibited by 35 min treatment with 1-NA-PP1 is very high. Note that inhibitory effects on pause release by unspecific inhibition of other kinases upon heat shock can be excluded with the use of the chemical biology (Shokat) approach, thus, inhibition of CDK9 is highly specific. Genome browser views of TT-seq signals of control (grey) and CDK9^{as} inhibited samples at the SESTD1 gene locus (163,098 [bp]) on chromosome 2 (b), at the CYB5R4 gene locus (107,781 [bp]) on chromosome 6 (c), and at the AGPS gene locus (151,192 [bp]) on chromosome 2 (d). Grey box depicts transcript body from TSS (black arrow) to polyA site (pA).

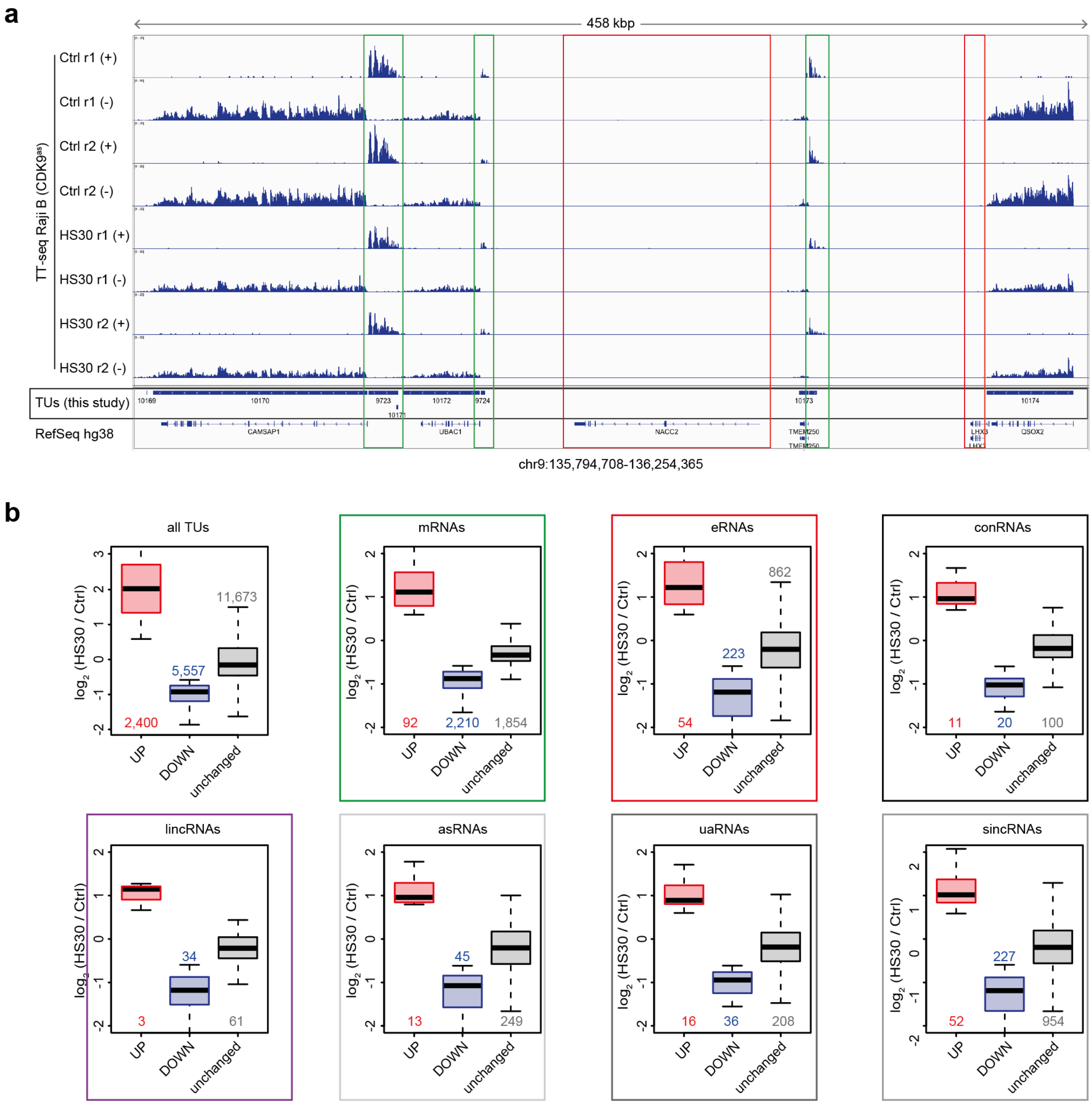

Supplementary Figure 9. **New GenoSTAN annotation of genes in Raji B (CDK9[as]) cells and differential expression.**

(a) Genome browser track of 458 kbp of chromosome 9 (chr9: 135,794,708-136,254,365) visualized with the Integrative Genomics Viewer (IGV, version 2.4.10)[6]. From top to bottom tracks represent: TT-seq coverage in Raji B (CDK9[as]) cells (n=4), new TU annotation (Methods), RefSeq GRCh38 annotation. TUs missing in the RefSeq annotation are highlighted in a green. RefSeq transcripts not present in Raji B cells are highlighted in red. (b) TUs and transcript classes as annotated in this study: all TUs, mRNAs (green frame), eRNAs (light red frame), conRNAs (black frame), lincRNAs (purple frame), asRNAs, uaRNAs, sincRNAs (different shades of grey). Boxplots show differential expression (DE) analysis of upregulated (light red), downregulated (dark blue), and unchanged TUs (grey) in human Raji B cells upon 30 min of heat shock. Heat shock samples (HS30) were compared to respective controls (Ctrl) of spike-in normalized TT-seq data. A minimum fold change of 1.5 in all conditions and a maximum adjusted P-value of 0.1 was set for calling a significant expression change.

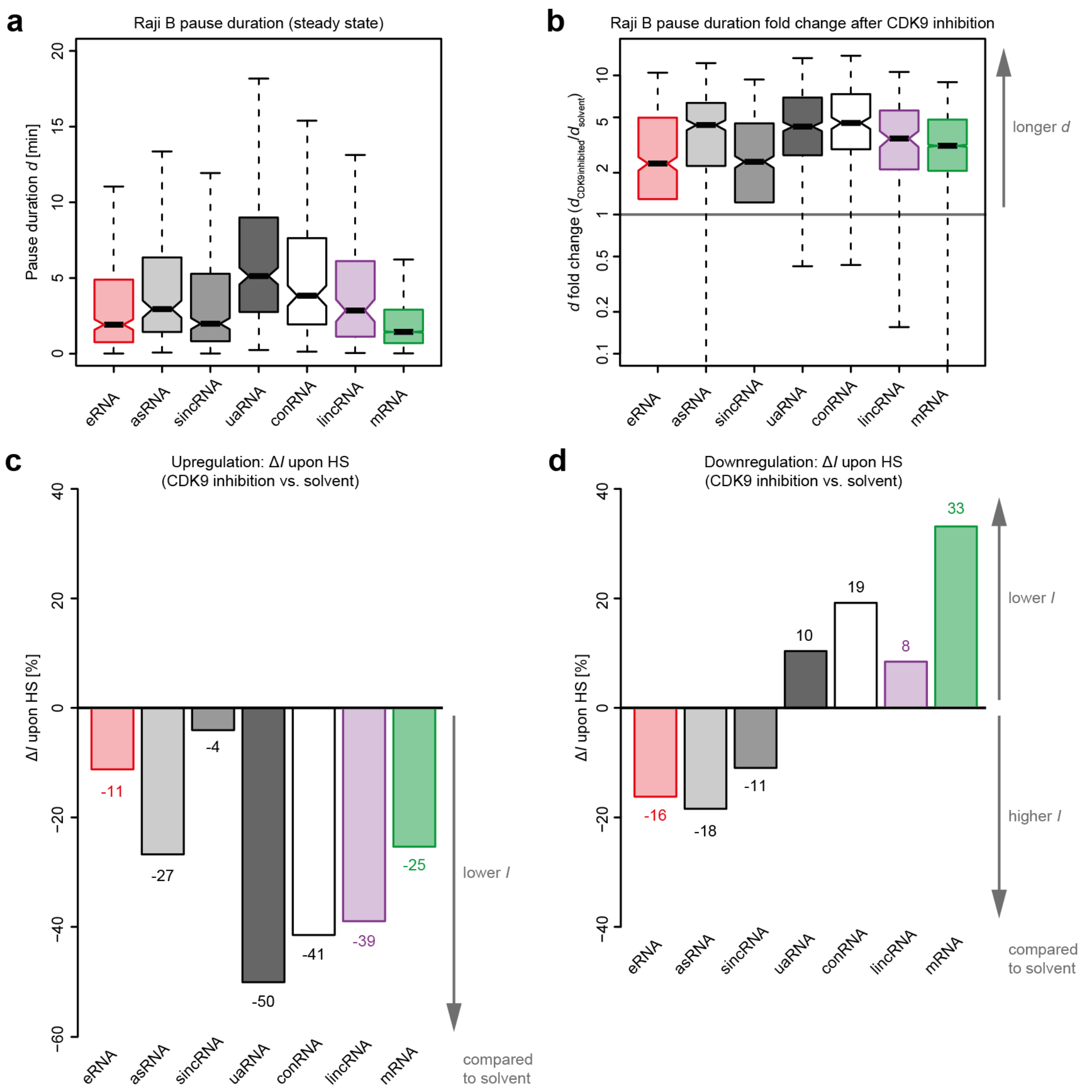

Supplementary Figure 10. **Gene classes vary in CDK9-induced pause duration changes upon heat shock.**

(a) Boxplots of pause duration *d* [min] for 359 eRNAs (in light red), 192 asRNAs, 365 sincRNAs, 311 uaRNAs (in different shades of grey), 179 conRNAs, 115 lincRNAs (in purple), and 5,768 mRNAs (in green) annotated in Raji B ($CDK9^{as}$) cells (Methods). By combining TT-seq data with published mNET-seq in Raji B ($CDK9^{as}$) cells [3], *d* was calculated. Black bars represent medians, boxes span from upper to lower quartiles, whiskers represent 1.5 times the inter-quartile range. Note that the median pause duration (black bars) per transcript class in Raji B ($CDK9^{as}$) cells resembles the median pause duration per transcript class in K562 cells (Fig. 2 c). Thus, our data suggest strong conservation across human hematopoietic cell lines. (b) Fold change of pause duration *d* for different transcript classes (colors as in (a) for 501 eRNAs, 318 asRNAs, 500 sincRNAs, 461 uaRNAs, 253 conRNAs, 145 lincRNAs, and 5,967 mRNAs) in Raji B ($CDK9^{as}$) cells upon CDK9 inhibition using published TT-seq and mNET-seq data[3]. CDK9 inhibited samples (5 µM 1-NA-PP1, 15 min) were compared to respective solvent control (DMSO, 15 min). Grey arrow indicates shift to longer pause durations upon CDK9 inhibition for all genes. (c) Bar plot comparing productive initiation frequency *I* change (Δ*I*) with and without CDK9 inhibition for significantly upregulated genes

in Raji B (CDK9^{as}) cells. From left to right: 54 eRNAs, 13 asRNAs, 52 sincRNAs, 16 uaRNAs, 11 conRNAs, 3 lincRNAs, and 92 mRNAs annotated in this study (colors as in (a)) (Supplementary Fig. 9 b; Methods). Δ*I* upon upregulation between heat shock with CDK9 inhibition (CDK9 inhibited HS30) and heat shock with solvent control (Solvent HS30) is shown in percent [%]. Grey arrow indicates that less productive initiation events occur compared to solvent control. (d) Bar plot comparing productive initiation frequency *I* change (Δ*I*) with and without CDK9 inhibition for significantly downregulated genes in Raji B (CDK9^{as}) cells. From left to right: 223 eRNAs, 45 asRNAs, 227 sincRNAs, 36 uaRNAs, 20 conRNAs, 34 lincRNAs, and 2,210 mRNAs annotated in this study (colors as in (a)) (Supplementary Fig. 9 b; Methods). Δ*I* upon downregulation between heat shock with CDK9 inhibition (CDK9 inhibited HS30) and heat shock with solvent control (Solvent HS30) is shown in percent [%]. Grey arrows indicate that less productive initiation events occur for mRNAs, or more productive initiation events for eRNAs (less downregulation) compared to solvent control.

Supplementary tables

Supplementary Table 1. **Information on experimental conditions used in this study.** Abbreviations used: control (Ctrl), heat shock (HS), analog sensitive CDK9 ($CDK9^{as}$).

No.	Assay	Cell type	Condition name	Replicate no.	Treatment
**1**	mNET-seq	K562	Ctrl	1	-
**2**	mNET-seq	K562	Ctrl	2	-
**3**	mNET-seq	K562	HS15	1	42 °C, 15 min.
**4**	mNET-seq	K562	HS15	2	42 °C, 15 min.
**5**	mNET-seq	K562	HS30	1	42 °C, 30 min.
**6**	mNET-seq	K562	HS30	2	42 °C, 30 min.
**7**	TT-seq	K562	Ctrl	1	-
**8**	TT-seq	K562	Ctrl	2	-
**9**	TT-seq	K562	HS15	1	42 °C, 15 min.
**10**	TT-seq	K562	HS15	2	42 °C, 15 min.
**11**	TT-seq	K562	HS30	1	42 °C, 30 min.
**12**	TT-seq	K562	HS30	2	42 °C, 30 min.
**13**	RNA-seq	K562	Ctrl	1	-
**14**	RNA-seq	K562	Ctrl	2	-
**15**	RNA-seq	K562	HS15	1	42 °C, 15 min.
**16**	RNA-seq	K562	HS15	2	42 °C, 15 min.
**17**	RNA-seq	K562	HS30	1	42 °C, 30 min.
**18**	RNA-seq	K562	HS30	2	42 °C, 30 min.
**19**	TT-seq	Raji B ($CDK9^{as}$)	Ctrl Solvent	1	0.05 % v/v DMSO, 35 min.
**20**	TT-seq	Raji B ($CDK9^{as}$)	Ctrl Solvent	2	0.05 % v/v DMSO, 35 min.
**21**	TT-seq	Raji B ($CDK9^{as}$)	Ctrl CDK9 inhibited	1	5 µM 1-NA-PP1, 35 min.

**22**	TT-seq	Raji B (CDK9^{as})	Ctrl CDK9 inhibited	2	5 µM 1-NA-PP1, 35 min.
**23**	TT-seq	Raji B (CDK9^{as})	HS Solvent	1	0.05 % v/v DMSO, 35 min; after 5 min: 42 °C, 30 min.
**24**	TT-seq	Raji B (CDK9^{as})	HS Solvent	2	0.05 % v/v DMSO, 35 min; after 5 min: 42 °C, 30 min.
**25**	TT-seq	Raji B (CDK9^{as})	HS CDK9 inhibited	1	5 µM 1-NA-PP1, 35 min; after 5 min: 42 °C, 30 min.
**26**	TT-seq	Raji B (CDK9^{as})	HS CDK9 inhibited	2	5 µM 1-NA-PP1, 35 min; after 5 min: 42 °C, 30 min.

Supplementary Table 2. **Sequencing statistics of 26 libraries generated in this study.**

All libraries were sequenced on a NEXTseq 550 sequencing platform in 75 bp paired-end mode. Numbers refer to experimental conditions listed in Supplementary Table 1. Correlation of replicates is calculated as Spearman's rho rounded to second decimal place.

**No.**	**Barcode**	**Fragment number**			**Duplicates (%)**	**Replicate correlation**
		**Sequenced**	**Mapped uniquely**	**Replicates combined**		
**1**	CGGAAT	159,190,513	118,817,488		54.1	
**2**	CTAGCT	173,587,084	130,654,902	249,472,390	54.5	1.00
**3**	GACGAC	164,964,729	117,933,814		48.0	
**4**	TAATCG	151,647,527	111,634,549	229,568,363	44.3	1.00
**5**	TCATTC	202,842,209	151,118,454		52.9	
**6**	TCCCGA	180,695,911	134,847,488	285,965,942	47.5	1.00
**7**	AAGCCT	149,673,334	134,367,932		7.2	
**8**	GTCGTA	151,253,343	136,351,777	270,719,709	11.5	1.00
**9**	AAGAGG	161,455,492	145,506,316		8.4	
**10**	GGAGAA	157,619,872	141,621,200	287,127,516	11.3	1.00
**11**	AGCATG	147,638,717	132,928,558		8.1	
**12**	GAGTCA	170,970,259	153,960,400	286,888,958	16.2	0.99
**13**	AACCAG	102,213,680	84,822,615		35.6	
**14**	TGGTGA	107,355,413	89,846,989	174,669,604	33.7	1.00
**15**	AGTGAG	100,709,060	84,896,024		35.4	
**16**	GCACTA	105,407,471	88,182,473	173,078,497	41.8	1.00
**17**	CACAGT	108,465,109	90,694,343		35.6	
**18**	TTGGCA	105,327,756	88,593,759	179,288,102	41.2	1.00
**19**	AACCAG	57,249,175	52,426,514		5.3	
**20**	TGGTGA	76,191,286	69,811,550	122,238,064	6.3	1.00

**21**	AGTGAG	70,801,390	64,421,316		15.1	
**22**	GCACTA	67,661,931	61,817,282	126,238,598	10.2	1.00
**23**	ACCTCA	70,180,248	64,629,409		6.5	
**24**	GTGCTT	74,373,094	68,428,032	133,057,441	8.5	1.00
**25**	AAGCCT	82,625,021	75,042,304		21.7	
**26**	GTCGTA	77,366,563	70,613,758	145,656,062	14.4	1.00

Supplementary Table 3. **List of qPCR primers for ACTB, HSP1A1, and synthetic RNA spike-ins used in this study.**

Spike-ins are derived from selected synthetic sequences of the ERCC Spike-in Mix (Methods).

Target name	Direction	Primer sequences	Length (nt)	T_m (°C)	Amplicon size (bp)	Genomic Sequence	Amplified Region	Position
RNA spike-in 4	forward	CCGAGTTCGC CTTACTGCTC	20	60	95	Synthetic, ERCC-00136	438 … 532	NA
	reverse	AATCGATCGG AATCACGCCG	20	60				
RNA spike-in 5	forward	CATAAGCGGA GAAAGAGGGA ATGAC	25	59	103	Synthetic, ERCC-00145	15 … 117	NA
	reverse	GCTAAATAGA GAGCATCCAC ACCTC	25	58				
RNA spike-in 12	forward	AGACTGGCAT TCCCGTGATA	20	57	97	Synthetic, ERCC-00170	222 … 318	NA
	reverse	GCTAAAACCC CTGCCTGCAA	20	60				
Actin beta (ACTB)	forward	ACTCTTCCAG CCTTCCTTCC	20	62	102	Gene ID: 60, NC_000007.14 Chromosome 7 Reference GRCh38.p12	2,299 … 2,495	exon
	reverse	TACAGGTCTT TGCGGATGTC	20	60				
Heat shock protein family A (Hsp70) member 1A (HSPA1A)	forward	GATCTTCACC ACCTACTCCG ACA	23	59	86	Gene ID: 3303, NC_000006.12 Chromosome 6 Reference GRCh38.p12	31,817,034 … 31,817,119	exon
	reverse	GATTGTTGTCT TTCGTCATGG CCC	24	60				

Supplementary Table 4. **List of IP antibody used in this study.**

Target	Host	Application	Dilution	Reference	Lot. #
anti-Pol II total CTD (unphos+phos)	mouse	mNET-seq	30 µg/IP	Biozol, MABI0601	15013

Supplementary Table 5. **List of previously published datasets used in this study.**

No.	Data	Cell line	Used for	Reference	Available at
1	Chromatin state annotation K562	K562	New annotation (cTU) of K562	Zacher et al. 2017 [8]	https://i12g-gagneurweb.in.tum.de/public/paper/GenoSTAN/
2	GRO-cap	K562	New annotation (cTU) of K562	Core et al. 2014 [9]	NCBI Gene Expression Omnibus (GSE60456)
3	H3K4me1	Raji B	New annotation (TU) of Raji B	Descostes et al. 2014 [10]	NCBI Gene Expression Omnibus (GSE52914)
4	H3K4me3	Raji B	New annotation (TU) of Raji B	Descostes et al. 2014 [10]	NCBI Gene Expression Omnibus (GSE52914)
5	mNET-seq	Raji B ($CDK9^{as}$)	Pause duration *d* of Raji B	Gressel et al. 2017 [3]	NCBI Gene Expression Omnibus (GSE96056)
6	TT-seq	Raji B ($CDK9^{as}$)	Pause duration *d* of Raji B	Gressel et al. 2017 [3]	NCBI Gene Expression Omnibus (GSE96056)
7	In situ Hi-C	K562	Revision Figure 3; Supplementary Figure 2 b	Rao et al. 2014 [11]	NCBI Gene Expression Omnibus (GSM1551620)
8	MNase-seq	K562	Revision Figure 3 b	Kundaje et al. 2012 [12]	NCBI Gene Expression Omnibus (GSE35586)
9	AT content	*in silico*	Revision Figure 3; Supplementary Figure 2 b	this study	NA
10	TOP1-seq	HCT116	Revision Figure 3; Supplementary Figure 2 b	Baranello et al. 2016 [13]	NCBI Gene Expression Omnibus (GSE57628)
11	Bisulfite-seq (RRBS)	K562	Revision Figure 3; Supplementary Figure 2 b	Myers et al. 2011 [14]	NCBI Gene Expression Omnibus (GSE27584)
12	CG content	*in silico*	Revision Figure 3; Supplementary Figure 2 b	this study	NA

13	CpG island	*in silico*	Revision Figure 3; Supplementary Figure 2 b	this study	NA
14	DNA-RNA melting temperature	*in silico*	Revision Figure 3; Supplementary Figure 2 b	this study	NA
15	DNase I hypersensitive sites (DHSs)	K562	Revision Figure 3 b	Crawford, Ohler et al. 2012 [15]	NCBI Gene Expression Omnibus (GSE32970)

Data accession links

1: https://i12g-gagneurweb.in.tum.de/public/paper/GenoSTAN/

2: https://www.ncbi.nlm.nih.gov/geo/query/acc.cgi?acc=GSE60456

3-4: https://www.ncbi.nlm.nih.gov/geo/query/acc.cgi?acc=GSE52914

5-6: https://www.ncbi.nlm.nih.gov/geo/query/acc.cgi?acc=GSE96056

7: https://www.ncbi.nlm.nih.gov/geo/query/acc.cgi?acc= GSM1551620

8: https://www.ncbi.nlm.nih.gov/geo/query/acc.cgi?acc= GSE35586

10: https://www.ncbi.nlm.nih.gov/geo/query/acc.cgi?acc= GSE57628

11: https://www.ncbi.nlm.nih.gov/geo/query/acc.cgi?acc= GSE27584

15: https://www.ncbi.nlm.nih.gov/geo/query/acc.cgi?acc= GSE32970

References

1. Mahat, D.B., Salamanca, H.H., Duarte, F.M., Danko, C.G. & Lis, J.T. Mammalian Heat Shock Response and Mechanisms Underlying Its Genome-wide Transcriptional Regulation. *Mol Cell* **62**, 63-78 (2016).
2. Ehrensberger, A.H., Kelly, G.P. & Svejstrup, J.Q. Mechanistic interpretation of promoter-proximal peaks and RNAPII density maps. *Cell* **154**, 713-715 (2013).
3. Gressel, S. et al. CDK9-dependent RNA polymerase II pausing controls transcription initiation. *Elife* **6** (2017).
4. Leppa, S., Kajanne, R., Arminen, L. & Sistonen, L. Differential induction of Hsp70-encoding genes in human hematopoietic cells. *J Biol Chem* **276**, 31713-31719 (2001).
5. Love, M.I., Huber, W. & Anders, S. Moderated estimation of fold change and dispersion for RNA-seq data with DESeq2. *Genome Biol* **15**, 550 (2014).
6. Robinson, J.T. et al. Integrative genomics viewer. *Nat Biotechnol* **29**, 24-26 (2011).
7. Ashburner, M. et al. Gene ontology: tool for the unification of biology. The Gene Ontology Consortium. *Nat Genet* **25**, 25-29 (2000).
8. Zacher, B. et al. Accurate Promoter and Enhancer Identification in 127 ENCODE and Roadmap Epigenomics Cell Types and Tissues by GenoSTAN. *PLoS One* **12**, e0169249 (2017).
9. Core, L.J. et al. Analysis of nascent RNA identifies a unified architecture of initiation regions at mammalian promoters and enhancers. *Nat Genet* **46**, 1311-1320 (2014).
10. Descostes, N. et al. Tyrosine phosphorylation of RNA polymerase II CTD is associated with antisense promoter transcription and active enhancers in mammalian cells. *Elife* **3**, e02105 (2014).
11. Rao, S.S. et al. A 3D map of the human genome at kilobase resolution reveals principles of chromatin looping. *Cell* **159**, 1665-1680 (2014).

12. Kundaje, A. et al. Ubiquitous heterogeneity and asymmetry of the chromatin environment at regulatory elements. *Genome Res* **22**, 1735-1747 (2012).

13. Baranello, L. et al. RNA Polymerase II Regulates Topoisomerase 1 Activity to Favor Efficient Transcription. *Cell* **165**, 357-371 (2016).

14. Consortium, E.P. An integrated encyclopedia of DNA elements in the human genome. *Nature* **489**, 57-74 (2012).

15. Natarajan, A., Yardimci, G.G., Sheffield, N.C., Crawford, G.E. & Ohler, U. Predicting cell-type-specific gene expression from regions of open chromatin. *Genome Res* **22**, 1711-1722 (2012).
