# Supplementary material for: The pause-initiation limit restricts transcription activation in human cells
Source: Nat Commun. 2019 Aug 9;10:3603. doi: 10.1038/s41467-019-11536-8 (PMC6689055; doi:10.1038/s41467-019-11536-8)
Supplement: Supplementary file 2 — Peer Review [file 41467_2019_11536_MOESM2_ESM.pdf]

Reviewers' comments:

Reviewer #1 (Remarks to the Author):

In this manuscript the authors have integrated information of nascent RNA synthesis rate and the RNA polymerase II (Pol II) density data to construct a kinetic model that could quantify the effect of Pol II pausing in transcriptional activation. They have combined TT-seq and mNET-seq data to show that "productive initiation frequency" of protein coding genes increase upon transcriptional activation with a decrease in pause duration. Activation of "pause-initiation limit" restricted genes is dependent on CDK9 kinase activity while transcription of enhancers are usually not restricted by "pause-initiation limit" and are less dependent on CDK9 for activation. Overall, this work confirms many of the previous findings that heat shock dependent gene activation is regulated at the level of paused Pol II release in Drosophila, humans and mice, which in turn is dependent on P-TEFb kinase activity. The authors present evidence that transcription directionality is determined by asymmetry in pause-release regulation between sense and anti-sense transcription. Intriguingly, the authors find that enhancer transcription, unlike transcription at protein-coding genes, does not appear to be regulated at the level of pause-release. Overall, the results presented both clarify and provide new detail concerning the essential role of promoter-proximal pausing in regulating transcription.

The manuscript is well written and experiments appear to be properly executed. Relevant datasets and critical metadata are clearly presented, and the authors demonstrate a high level of reproducibility across biological replicates in all cases. The authors' use of a rigorous RNA spike-in normalization strategy allows for global changes in transcription to be measured, which is absolutely essential and yet often lacking in the literature. Data processing and analysis appear to our eyes to be likewise rigorous, and we find no obvious fault in the equations used for kinetic modeling.

While we have provided some questions, concerns, and recommendations for the authors below, we would nonetheless recommend acceptance of this manuscript after addressing these points and without a requirement for additional experiments.

Comments:
1. Is it possible that the half-lives of enhancer RNAs are short enough to interfere with kinetic parameter estimates? If so, perhaps some qualifying statement should be made.
2. The authors report that following heat shock the fold change of initiation frequency is higher for upregulated enhancers than promoters that have the same fold-change in pause duration. There are enhancers that show heat shock induced binding of the master regulator HSF1 but most do not (Vihervaara et al 2017). It would be interesting to know if the classes of upregulate enhancers and promoters that are driven by HSF1 show this same difference. This might address if enhancer and promoters that share a common architecture and a common regulatory factor can behave so differently.
3. The authors have mentioned that eRNAs do not adopt stable secondary structure (-15 to -65 from pause site) and that could be a reason for their low pause durations however, the downregulation of eRNA transcription follows similar pattern to that seen for mRNA downregulation. This is a very interesting but limited observation and it would be good to see if other chromatin features, nucleosome positioning or density could provide any insights into this differential property of upregulated and downregulated enhancers.
4. The authors propose a "model" in which the upregulation of enhancer transcription precedes a decrease in pause duration of protein coding genes without any mechanistic detail or evidence for the temporal relationship. We suspect this is simply a technical miswording.
5. Recovery of in vitro transcribed and thio-uridine RNA is used to infer cellular recovery of labeled RNA in TT-seq, and it looks like the assumption is 10% of nascent RNA uridines will be thiolated. Please cite from where this assumption comes.
6. The text states that downregulated enhancers were not affected by Cdk9 inhibition (Fig4C; Sup. 10D), but it looks like percent repression increased.

7. Throughout the main text, the meaning of "initiation" in the context of the kinetic modeling is clear. The authors might consider making the meaning explicit in the discussion section, as this could be confusing for readers.
8. The concluding sentence of the section "Cdk9 activity lowers the pause-initiation limit for gene activation", line 195, seems to be referencing the results covered in the next section.
9. Figures 4C, etc. are labeled in percentages, but display proportions/decimal fractions.
10. In Figure 4D-E, it would be nice if the relevant cell line for each panel were apparent within the figure itself.

Typos and Grammar, in page order
1. Grammar in line 32 (last sentence of first paragraph). Possible rewording: "However, whether and to what extent pausing can restrict..."
2. Wording in lines 37. Instead of mapping Pol II "along" RNA, might consider wording as "using RNA" for clarity.
3. Line 60, there should not be a comma after "limit".
4. Line 112, the word "and" is missing.
5. Lines 201-202 reference
6. In methods section lines 400-407 (sequencing depth and cross-contamination rate), the variable isn't explicitly defined.
7. In methods line 612, the word "below" should probably be changed to "less than".

Reviewer #2 (Remarks to the Author):

This essentially bioinformatic analysis compares Pol II occupancy derived from mNET-seq libraries to productive transcriptional initiation/elongation based on TT-seq. In effect this study aims to quantitate how many polymerases pause out of those that productively transcribe across genes.
This study is clearly an interesting addition to our understanding of TSS proximal pausing by Pol II. However I feel a direct comparison with previous work as below would be useful.

Previous analyses such as Schlackow et al. (2017) measure the "Escaping Index" to determine how many polymerases paused in TSS proximal regions actually proceed to elongate. This type of analysis reveals how many polymerases accumulate over the TSS proximal pause region, but possibly this is different to the measure of pausing (pause initiation limit-PIL) as presented in this study. Essentially the Escaping index includes polymerases that actually terminate over the pause region together with those that escape into productive elongation into the gene body. Presumably PIL quantitation only considers polymerases that are ultimately productive. This may explain why protein coding and lincRNA pausing look the same, in conflict with previous analysis. It should also be considered that lncRNA transcription is much more prone to premature termination than protein coding transcription (Schlackow et al. 2017)
Really this study needs to directly compare the PIL method of estimating pausing with the previously employed escape index approach. Also, I wonder about the validity of the lncRNA TU annotations based on GenoSTAN
as lncRNA need careful independent definition based on nascent transcription profile.

Specific comments

1. Which CTD modifications were used for the mNET-Seq data? To measure TSS pausing the use of unphosphorylated or Y1P CTD would be best.
2. It is claimed that the index derived from TT-Seq measures the number of initiating polymerases. However, in Sup Figure 5e it is evident that TT-Seq has a lot more reads over exons, so possbkle it is contaminated by steady state RNA? Is this frequently the case for TT-Seq? This may lead to a problem in normalization, as lincRNAs are mainly degraded in the nucleoplasm.
3. It would be informative to show metagene profiles for TT-Seq and mNET-Seq for the different Pol II TU classes (protein coding, linRNA, eRNA).
4. There is some concern that heat-shock activation may generate a lot of indirect effects and so confound some of this transcriptional analysis.

<u>Point-by-point responses to reviewers' comments</u>

Nature Communication research article NCOMMS-19-02193-T

The pause-initiation limit restricts transcription activation in human cells

by Saskia Gressel, Björn Schwalb, and Patrick Cramer

Responses are in italics

We thank the referees for their thoughtful and diligent review of our manuscript. In the revised manuscript we have made every effort to respond to the reviewers' concerns, and in so doing, we believe we have strengthened and extended our main conclusions.

Reviewer #1:

In this manuscript the authors have integrated information of nascent RNA synthesis rate and the RNA polymerase II (Pol II) density data to construct a kinetic model that could quantify the effect of Pol II pausing in transcriptional activation. They have combined TT-seq and mNET-seq data to show that "productive initiation frequency" of protein coding genes increase upon transcriptional activation with a decrease in pause duration. Activation of "pause-initiation limit" restricted genes is dependent on CDK9 kinase activity while transcription of enhancers are usually not restricted by "pause-initiation limit" and are less dependent on CDK9 for activation. Overall, this work confirms many of the previous findings that heat shock dependent gene activation is regulated at the level of paused Pol II release in Drosophila, humans and mice, which in turn is dependent on P-TEFb kinase activity. The authors present evidence that transcription directionality is determined by asymmetry in pause-release regulation between sense and anti-sense transcription. Intriguingly, the authors find that enhancer transcription, unlike transcription at protein-coding genes, does not appear to be regulated at the level of pause-release. Overall, the results presented both clarify and provide new detail concerning the essential role of promoter-proximal pausing in regulating transcription.

The manuscript is well written and experiments appear to be properly executed. Relevant datasets and critical metadata are clearly presented, and the authors demonstrate a high level of reproducibility across biological replicates in all cases. The authors' use of a rigorous RNA spike-in normalization strategy allows for global changes in transcription to be measured, which is absolutely essential and yet often lacking in the literature. Data processing and analysis appear to our eyes to be likewise rigorous, and we find no obvious fault in the equations used for kinetic modeling.

We thank the reviewer for the kind words and appreciation.

While we have provided some questions, concerns, and recommendations for the authors below, we would nonetheless recommend acceptance of this manuscript after addressing these points and without a requirement for additional experiments.

Comments:

1) Is it possible that the half-lives of enhancer RNAs are short enough to interfere with kinetic parameter estimates? If so, perhaps some qualifying statement should be made.

*This is an important point and we have performed additional analyses to demonstrate that our kinetic parameter estimates are unbiased. The reviewer is right that this could interfere with our kinetic parameter estimates, as we derive the pause duration d from a combination of mNET-seq and TT-seq data. Thus, d is proportional to the ratio of mNET-seq signal over the productive initiation frequency I. If transcripts are degraded rapidly this would indeed lead to an underestimation of I which corresponds to the TT-seq signal of the respective transcripts. The denominator (I) would be smaller, and the inferred pause duration d would be longer. To control if our kinetic parameter estimates might be biased by the short half-lives, we calculated half-lives of eRNAs from TT-seq and RNA-seq data in K562 cells for instances with long and short pause durations (**Response Figure 1**). As no significant effect is visible, we conclude that half-lives of eRNAs do not interfere with our kinetic measurements. Note also that, in the case of a stability induced bias the derived pause duration for eRNAs would be overestimated. The real pause duration estimate would thus be even further away from the pause initiation limit and this would even strengthen our conclusions further.*

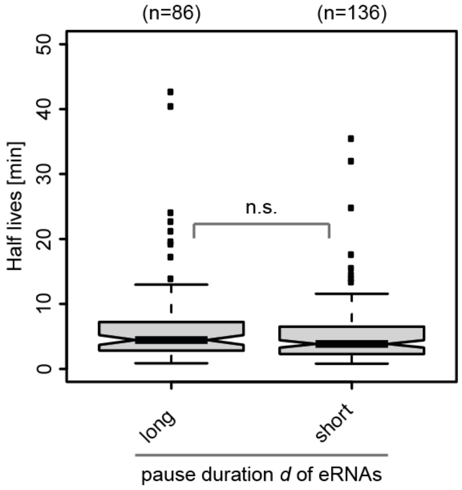

Response Figure 1. Pause durations of eRNAs are not biased by the short half-lives of eRNAs.

Kinetic modeling of TT-seq and RNA-seq in K562 cells allows us to estimate half-lives of different transcript classes[1,2]. Half-life estimates are depicted for eRNAs with long (n=68) and short pause durations (n=136). The comparison of both sets is not significant (n.s.; Wilcoxon rank sum test, p-value = 0.1528). Black bars represent medians, boxes represent upper and lower quartiles, and whiskers represent 1.5 times the interquartile range.

2) The authors report that following heat shock the fold change of initiation frequency is higher for upregulated enhancers than promoters that have the same fold-change in pause duration. There are enhancers that show heat shock induced binding of the master regulator HSF1 but most do not (Vihervaara et al 2017). It would be interesting to know if the classes of upregulated enhancers and promoters that are driven by HSF1 show this same difference. This might address if enhancer and promoters that share a common architecture and a common regulatory factor can behave so differently.

We thank the reviewer for this thoughtful suggestion. We included the results in ***Response Figure 2****, the main text, methods as well as in* ***Figure 4 d****. We are using human cancer cell lines in this study and it has been established that HSF1 shows different chromatin binding in cancer*[3]*. Thus, we only considered HSF1 binding events that were enriched in heat shock conditions of cycling K562 cells (Vihervaara et al.*[4]*, data availability: GSE43579). When focusing only on HSF1 driven mRNAs and eRNAs (TSS proximal HSF1 peak calls,* ***Methods****) we observed an even clearer distinction between promoter-proximal pause regulated upregulation. HSF1 driven eRNAs can be activated without a change in pause duration, while HSF1 driven mRNAs still require a shortening of the pause duration (****Response Figure 2 b****). The enrichment of HSF1 at upregulated enhancers agrees with a study by Vihervaara et al.*[5]*.*

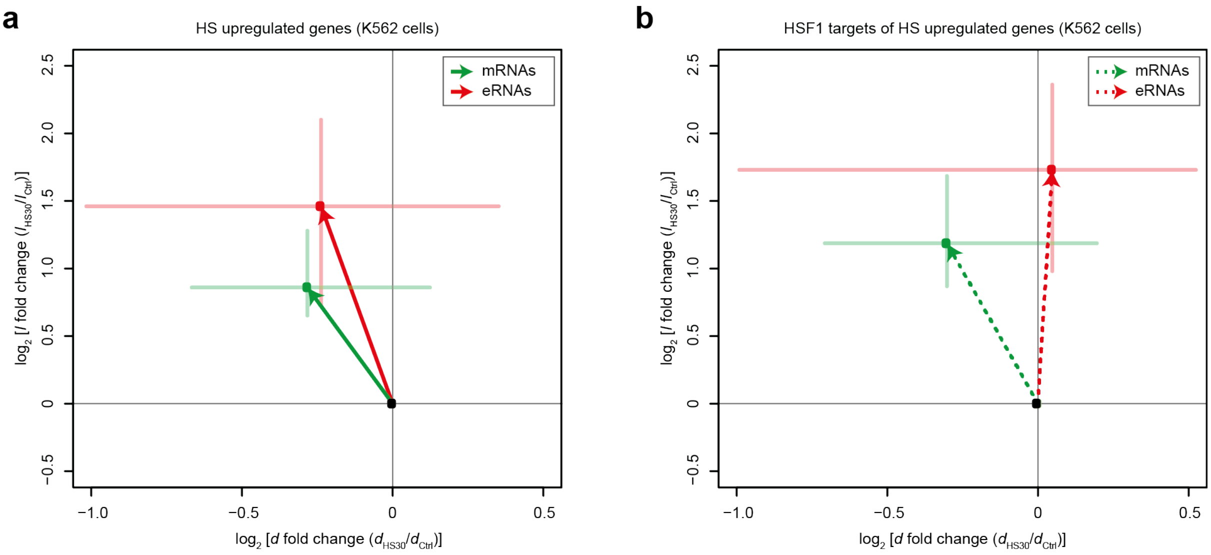

Response Figure 2. HSF1 driven enhancers can be upregulated without change in pause duration.

*Left panel (original manuscript, Figure 4 d): Log2 fold change of pause duration d and initiation frequency I for 336 significantly upregulated mRNAs (in green), and 70 significantly upregulated eRNAs (in red) in K562 cells upon 30 min of heat shock (HS30). Right panel (addition upon reviewer comment): Log2 fold change of pause duration d and initiation frequency I for 91 HSF1 driven (****Methods****) significantly upregulated mRNAs (in green), and 20 HSF1 driven significantly upregulated eRNAs (in red) in K562 cells upon 30 min of heat shock (HS30).*

3) The authors have mentioned that eRNAs do not adopt stable secondary structure (-15 to -65 from pause site) and that could be a reason for their low pause durations however, the

downregulation of eRNA transcription follows similar pattern to that seen for mRNA downregulation. This is a very interesting but limited observation and it would be good to see if other chromatin features, nucleosome positioning or density could provide any insights into this differential property of upregulated and downregulated enhancers.

*We now compared all transcript classes in steady-state K562 cells with respect to other (epi)genomic features (**Response Figure 3 a**, added to **Supplementary Figure 2 b**). To this end we used in silico analyses of the sequence content and published datasets which we added to the list in **Supplementary Table 5** (row 7-15). These (epi)genomic features differ from the data sets which we used for enhancer/promoter classification in our annotation (see **Methods**, and our response to the second reviewer, line 313-315). In the metagene analysis, enhancers and protein-coding genes show opposite behavior for the (epi)genomic features studied here: DNA-RNA melting temperature, sequence composition (CG/AT content), Bisulfite-seq[6] (detecting hypermethylated CpG islands), TOP1-seq[7] (detecting catalytically engaged topoisomerase I), and Hi-C[8] (detecting long-range chromatin interactions) (**Response Figure 3 a; Supplementary Figure 2 b**). However, these data are correlative in nature and require further experimental validation. If there are links between genomic context and the pause duration one would need to test this in future studies.*

*As suggested by the reviewer, we also added a comparison of chromatin features in steady-state for sets of heat shock (HS) up- and downregulated enhancers (**Response Figure 3 b**). A recent study showed that upon 30 min of HS no global changes in compartments (TADs) or looping interactions were observed in human K562 cells[9], indicating that Hi-C data in steady-state K562 cells may be used for comparison. However, on the chromatin accessibility level changes were observed upon HS in Drosophila cells[10]. Specifically, nucleosome loss at activated loci[11] and chromatin modification changes (i.e.. increased acetylation of H4[5]) in human cells. Thus, DNase-seq and MNase-seq signals may differ upon HS. It was also shown that topoisomerase 1 (TOP1) activity is changed upon HS in human cells[12]. In summary, suggested links between genomic context (chromatin interactions, hypermethylated CpG islands, TOP1 activity, accessibility) and sets of up- or downregulated enhancers would need to be tested in future studies.*

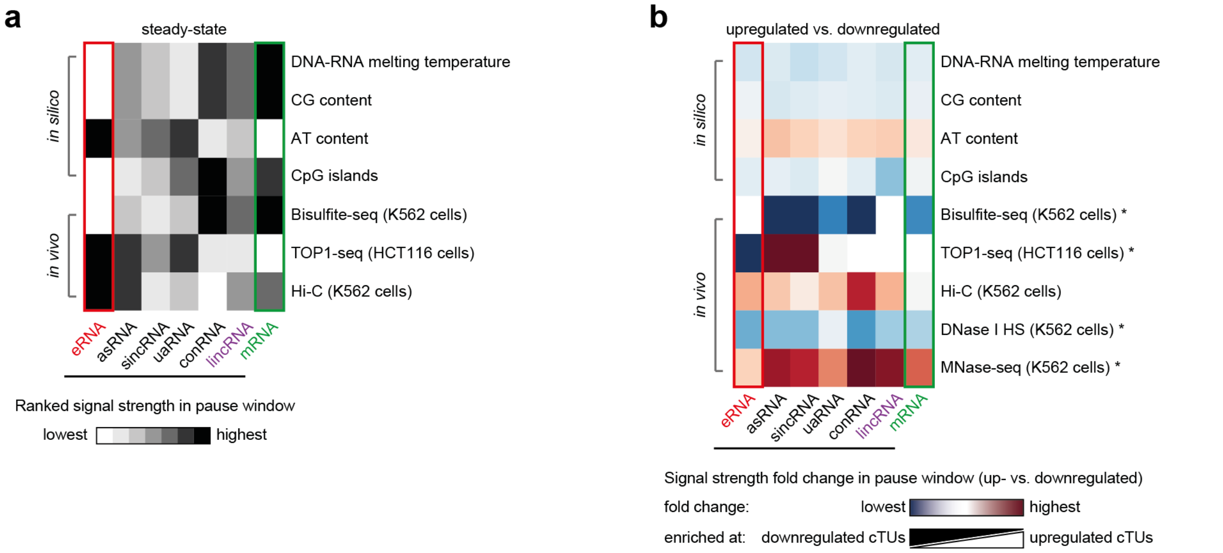

Response Figure 3. Chromatin features of different transcript classes.
*(a) Chromatin features in the pause window (**Methods**) of different transcript classes. Data are ranked by each row across different transcript types to highlight the contrast of individual features. Published data sets are listed in **Supplementary Table 5**. (b) Occupancy fold change in pause window of different chromatin features at significantly upregulated versus downregulated transcript types upon heat shock (HS). Changes of more than 2-fold do not increase in color intensity to rid color assignment of outliers. Note that certain (epi)genomic features might differ upon HS (marked by asterisk).*

4) The authors propose a "model" in which the upregulation of enhancer transcription precedes a decrease in pause duration of protein coding genes without any mechanistic detail or evidence for the temporal relationship. We suspect this is simply a technical miswording.

We thank the reviewer for pointing this out and apologize for the misworded conclusion in our discussion. We changed the wording to reflect the reviewer's concern. It is now clear from the statement, that this is simply a hypothetical model.

5) Recovery of in vitro transcribed and thio-uridine RNA is used to infer cellular recovery of labeled RNA in TT-seq, and it looks like the assumption is 10% of nascent RNA uridines will be thiolated. Please cite from where this assumption comes.

In general, a single 4sU residue would be sufficient to purify the labeled RNA spike-ins. In human cell lines, 4sU incorporation rates of 1-4% were observed [13-16]. To ensure at least similar 4sU incorporation rates in the IVT of the 4sU-labeled RNA spike-ins, we fixed this rate to 10%. We added a sentence to the methods section to reflect this.

6) The text states that downregulated enhancers were not affected by Cdk9 inhibition (Fig4C; Sup. 10D), but it looks like percent repression increased.

We changed the text accordingly.

7) Throughout the main text, the meaning of "initiation" in the context of the kinetic modeling is clear. The authors might consider making the meaning explicit in the discussion section, as this could be confusing for readers.

We have made sure 'initiation' is correctly understood as the initiation of RNA chain formation.

8) The concluding sentence of the section "Cdk9 activity lowers the pause-initiation limit for gene activation", line 195, seems to be referencing the results covered in the next section.

We double-checked and feel that this sentence concludes the paragraph well and would like to keep the text as is.

9) Figures 4C, etc. are labeled in percentages, but display proportions/decimal fractions.

Fixed.

10) In Figure 4D-E, it would be nice if the relevant cell line for each panel were apparent within the figure itself.

Fixed.

Typos and Grammar, in page order:

- Grammar in line 32 (last sentence of first paragraph). Possible rewording: "However, whether and to what extent pausing can restrict..."
- Wording in lines 37. Instead of mapping Pol II "along" RNA, might consider wording as "using RNA" for clarity.
- Line 60, there should not be a comma after "limit".
- Line 112, the word "and" is missing.
- Lines 201-202 reference
- In methods section lines 400-407 (sequencing depth and cross-contamination rate), the variable isn't explicitly defined.
- In methods line 612, the word "below" should probably be changed to "less than".

We changed text accordingly.

Reviewer #2:

This essentially bioinformatic analysis compares Pol II occupancy derived from mNET-seq libraries to productive transcriptional initiation/elongation based on TT-seq. In effect this study aims to quantitate how many polymerases pause out of those that productively transcribe across genes. This study is clearly an interesting addition to our understanding of TSS proximal pausing by Pol II.

We are glad that the reviewer finds our kinetic dissection of the heat shock response interesting. We would like to kindly disagree that this study is an 'essentially bioinformatic analysis'. Note, our multi-omics approach is based on 26 novel libraries (mNET-seq, TT-seq and RNA-seq) as well as additional experimental data.

However, I feel a direct comparison with previous work as below would be useful. Previous analyses such as Schlackow et al. (2017) measure the "Escaping Index" to determine how many polymerases paused in TSS proximal regions actually proceed to elongate. This type of analysis reveals how many polymerases accumulate over the TSS proximal pause region, but possibly this is different to the measure of pausing (pause initiation limit-PIL) as presented in this study. Essentially the Escaping index includes polymerases that actually terminate over the pause region together with those that escape into productive elongation into the gene body.

We agree that the observed Pol II peaks close to mammalian promoters measured by mNET-seq (in our study and in Schlackow et al.[17]) could be explained by rapidly initiating and then terminating polymerases (promoter-proximal attenuation), and we have pointed this out in the text. Unfortunately, there is no method available to distinguish attenuation from long pause duration. None of the Pol II occupancy methods (incl. mNET-seq), not even short capped RNA assays are able to distinguish the frequency of these events. Short capped RNA measurements are as well biased by the residence time of the polymerases since the nascent RNA associated with the elongation complex is longer protected (and thus, measurable) for loci with longer pause durations. We wish to emphasize that the events upstream of the pause site are irrelevant to our model and its conclusions. In addition, the pause duration d obtained in our model reflects the effective pause between two initiation events that successfully lead to productive elongation of a transcript and thus the relevant transcriptional outcome. This does not necessarily entail that one polymerase is paused the entire time estimated, it could also mean that a subpopulation of polymerases undergoes early termination at the pause site.

Presumably PIL quantitation only considers polymerases that are ultimately productive.

Our multi-omics approach combines TT-seq and mNET-seq measurements. TT-seq indeed provides the productive initiation frequency. On the other hand, mNET-seq measures all polymerases, independently of their activity/productivity.

This may explain why protein coding and lincRNA pausing look the same, in conflict with previous analysis. It should also be considered that lncRNA transcription is much more prone to premature termination than protein coding transcription (Schlackow et al. 2017). Really this

study needs to directly compare the PIL method of estimating pausing with the previously employed escape index approach.

We believe this is a misunderstanding. Schlackow et al.[17] *draw conclusions on Pol II pausing based on promoter escape indices derived from Pol II occupancies at mRNAs and lincRNAs (measured by mNET-seq). The authors conclude that lincRNAs have a lower promoter escape index compared to mRNAs, but their data cannot result in kinetic insights because they measure occupancy. Occupancy data cannot inform on the pause duration since the Pol II occupancy (mNET-seq signal) is determined both by the number of polymerases as well as their residence time*[18]*. This is a key point of our manuscript and the reason why we use multi-omics to uncover kinetic principles for regulation.*

The limitations of occupancy profiling were pointed out in ***Supplementary Note 1*** *and* ***Supplementary Figure 6****. However, for clarity, we added an additional figure for the respective transcription classes to highlight the bias of occupancy read-outs (in comparison to our multi-omics approach) (****Response Figure 4****, now added also to* ***Supplementary Figure 6****). If we compare the mNET-seq signal of mRNAs and lincRNAs in the promoter-proximal window (****Response Figure 4 a-b****), we observe smaller occupancy peaks in the case of lincRNAs which agrees with Schlackow et al.* [17]*. But, only when factoring in the number of productive initiation events measured by TT-seq, kinetic conclusions can be drawn (****Response Figure 4 a-b****). mRNAs show median productive initiation events of 1 Pol II cell^{-1}min^{-1} while lincRNAs initiate less (0.3 Pol II cell^{-1}min^{-1}). In addition, we disagree that Schlackow et al. provided definite proof that long noncoding (lnc) RNAs are more prone to premature termination. As discussed above, occupancy profiling cannot distinguish long pause duration from attenuation.*

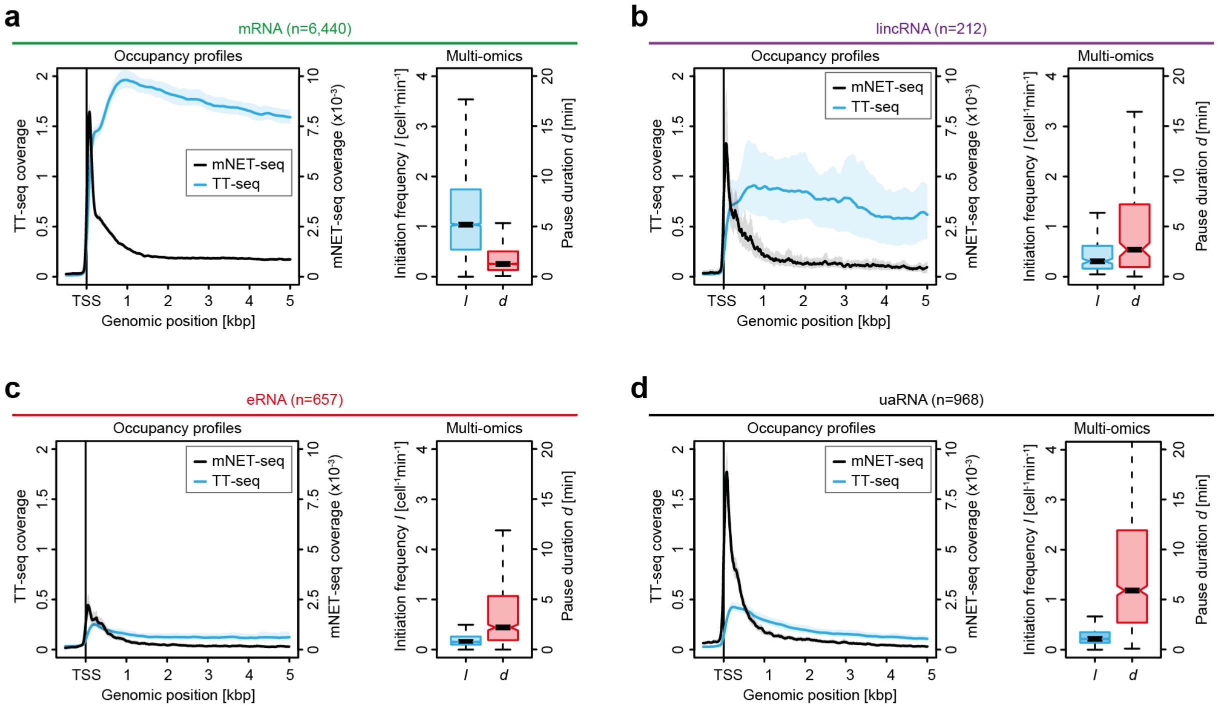

Response Figure 4. Multi-omics, but not occupancy profiling alone, can reveal transcription kinetics.

For comparison among different transcript classes, TT-seq coverage (left y-axis) and mNET-seq coverage (right y-axis) show the same range for all metagene profiles. Coverage per cell is shown for two biological replicates of steady-state K562 cells. (a-b) mRNAs (6,440, green) and lincRNAs (212, purple) show similar total Pol II peak heights in mNET-seq (with Empigen BB). However, the TT-seq signal is significantly lower for lincRNAs indicating less productive initiation events compared to mRNAs. (c-d) eRNAs (657, red) and uaRNAs (968, black) show a similar height of the TT-seq signal. However, Pol II occupancy strongly varies in these transcript classes. As a consequence, our multi-omics approach shows that pause durations are longer for uaRNAs than for eRNAs.

Also, I wonder about the validity of the lncRNA TU annotations based on GenoSTAN as lncRNA need careful independent definition based on nascent transcription profile.

We assume that the reviewer missed the information in the manuscript that our GenoSTAN-derived annotation is indeed based on newly synthesized transcripts (measured by TT-seq). We assume the reviewer uses 'nascent' (which means polymerase-associated RNA) to refer to 'newly synthesized' RNA (which may not be polymerase-associated anymore, i.e. for TT-seq, newly synthesized during a 5 min labeling pulse). In order to convince the reviewer of the validity of our transcript class annotation and classification – including lncRNA – we will give an additional detailed explanation in the following. As a validity criterion, all GenoSTAN-derived transcripts are required to have a 5' cap in their nascent (polymerase-associated) transcript (measured by GRO-cap[19]) to define expressed transcribed units (TUs) in our annotation. These expressed TUs are further classified using the GRCh38/hg20 RefSeq annotation of mRNAs and lincRNAs. (Upstream) antisense and convergent RNAs (uaRNAs, asRNAs, conRNAs) are defined with respect to the sense TSS of mRNAs or lincRNAs (see ***Methods****) while the remaining transcripts are classified as small intergenic noncoding (sinc) RNAs. All noncoding transcript classes (except lincRNAs) are then subjected to chromatin state annotation. In K562 cells we used 18 chromatin states to define promoter and enhancer states considering DNase-seq as well as ChIP-seq of 8 histone modifications (H3K27me3, H3K36me3, H4K20me1, H3K4me1-3, H3K27ac, H3K9ac) and of the histone acetyltransferase P300 (for details see[20]). If noncoding transcript classes fall in enhancer states, their instable product is referred to as putative enhancer RNA (eRNA). To date, it remains a significant challenge to accurately annotate sequences as promoter or enhancer[21,22]. We acknowledge that additional characterization by reporter gene analysis or CRISPR/Cas9 deletion experiments would provide a definite read-out of enhancer functionality. However, this would be beyond the scope of this study. In summary, GenoSTAN provides a powerful tool to annotate entire TUs genome-wide. In combination with start site refinement (e.g. by GRO-cap or PRO-cap) it allows for a very accurate, cell line specific annotation of TUs. We trust this clarifies the concern of the reviewer.*

Specific comments

1) Which CTD modifications were used for the mNET-Seq data? To measure TSS pausing the use of unphosphorylated or Y1P CTD would be best.

We agree with the reviewer that this is a critical point. In the submitted manuscript we therefore provided this information on the POLR2A (human Rpb1) antibody already in the ***Methods*** *section and in* ***Supplementary Table 4****. For kinetic modeling, it is critical to estimate the total number of Pol II molecules on a gene. We used an antibody which targets the total CTD (unphoshorylated and phosphorylated) of the POLR2A subunit of Pol II. This antibody is well established for profiling total Pol II in human cells (see Stasevich et al.[23]; Nojima et al.[24,25]).*

2) It is claimed that the index derived from TT-Seq measures the number of initiating polymerases. However, in Sup Figure 5e it is evident that TT-Seq has a lot more reads over exons, so possbkle it is contaminated by steady state RNA? Is this frequently the case for TT-Seq? This may lead to a problem in normalization, as lincRNAs are mainly degraded in the nucleoplasm.

*We agree with the reviewer that this is a critical point. This issue had been carefully addressed and corrected for in the submitted version of the manuscript. We summarize this again below. TT-seq measures newly synthesized RNA during a 5 min labeling pulse. As splicing events occur mainly co-transcriptionally, this can lead to higher signal in exons compared to introns. On the other hand, cross-contamination of pre-existing matured RNA might lead to a similar effect as this would also mainly contribute signal to exons. As mentioned by the reviewer, cross-contamination can be a critical bias. However, this can be assessed and controlled for by our (un)labeled spike-ins (****Methods****,* ***Response Figure 5****). The cross-contamination rate derived from our external control spike-ins give reliable per sample estimates of the fraction of contaminating unlabeled RNA that is found in the labeled RNA fraction and can be corrected for in the TT-seq signal. In general, we observed very low cross-contamination rates in our TT-seq experiments - in the range of 0.4 to 1 % when following the original protocol[2] and using HPDP-biotin. However, we are still correcting our TT-seq data for this by the cross-contamination rate described in detail in the* ***Methods*** *section. Thus, the difference observed in the cross-contamination corrected TT-seq signal at the EGR1 locus in* ***Supplementary Figure 5 e*** *is due to co-transcriptional splicing.*

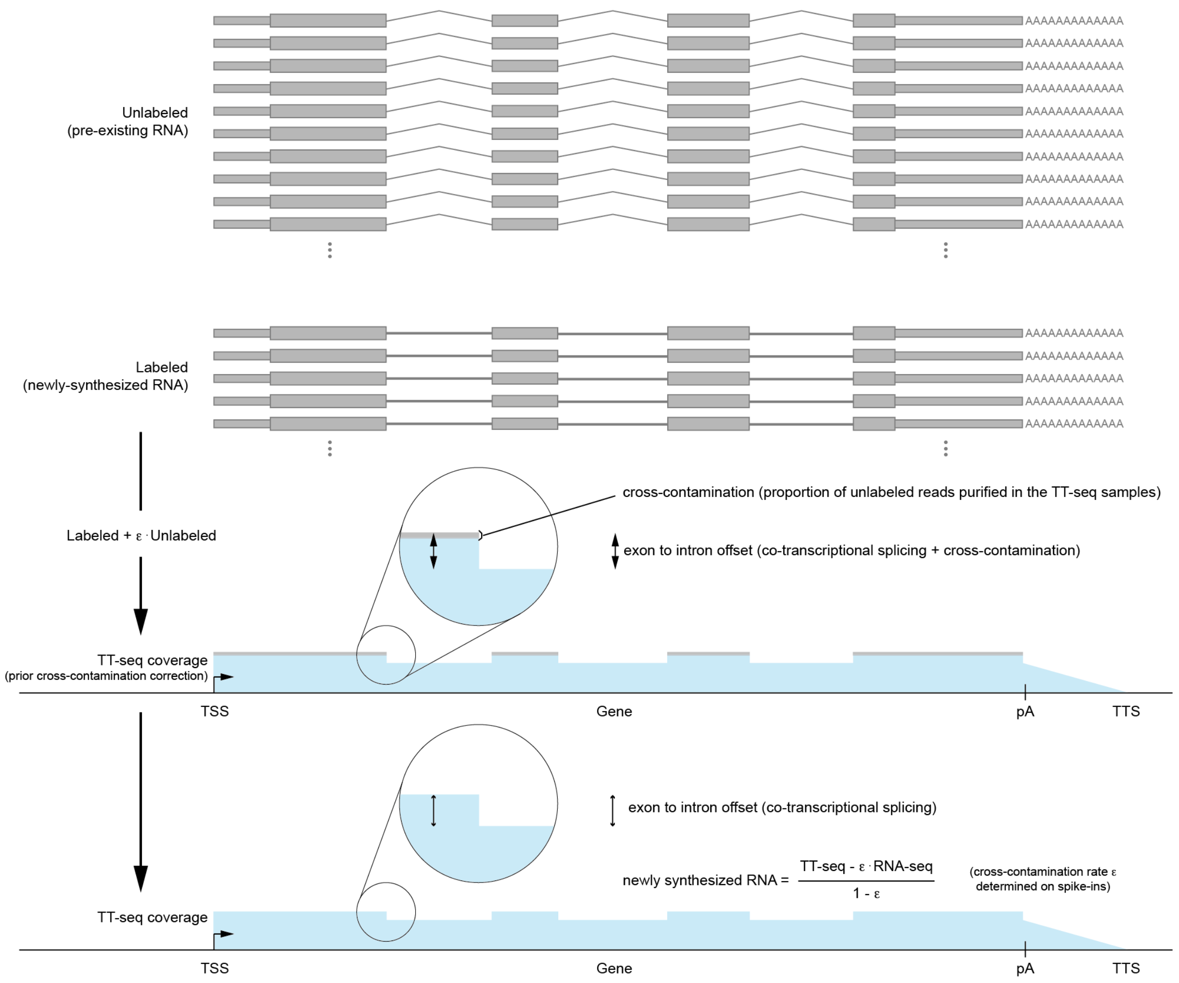

Response Figure 5. TT-seq signal shows exon-intron offset due to co-transcriptional splicing. *See main text for details.*

3) It would be informative to show metagene profiles for TT-Seq and mNET-Seq for the different Pol II TU classes (protein coding, linRNA, eRNA).

*This has already been addressed above. Please refer to the detailed responses for reviewer 1 related to **Response Figure 4**.*

4) There is some concern that heat-shock activation may generate a lot of indirect effects and so confound some of this transcriptional analysis.

*As mentioned in the introduction, the heat shock response was chosen as a case study of transcriptional regulation because it is a well-established model system. The characteristics of the HSR are well known and include a high induction of heat shock genes. In addition, cells shift all resources from growth to combat stress and ensure survival which involves the global shut-down of transcription of growth-related genes. Although one can never completely rule out an indirect effect of a treatment in vivo, the GO analysis supports that HS responsive genes are upregulated and growth-related genes are downregulated (**Supplementary Figure 7**). In addition, our multi-omics analysis is carried out at a temperature defined as 'mild HS' (see*

Shalgi et al. [26]*) and at early time points (15 and 30 min). This is possible because TT-seq quantifies even short-lived RNAs. Furthermore, TT-seq is particularly powerful in monitoring downregulation of transcription because it is not biased by stable transcripts which might mask rapid changes (in contrast to RNA-seq).*

References

1 Miller, C. *et al.* Dynamic transcriptome analysis measures rates of mRNA synthesis and decay in yeast. *Mol Syst Biol* **7**, 458, doi:10.1038/msb.2010.112 (2011).

2 Schwalb, B. *et al.* TT-seq maps the human transient transcriptome. *Science* **352**, 1225-1228, doi:10.1126/science.aad9841 (2016).

3 Mendillo, M. L. *et al.* HSF1 drives a transcriptional program distinct from heat shock to support highly malignant human cancers. *Cell* **150**, 549-562, doi:10.1016/j.cell.2012.06.031 (2012).

4 Vihervaara, A. *et al.* Transcriptional response to stress in the dynamic chromatin environment of cycling and mitotic cells. *Proc Natl Acad Sci U S A* **110**, E3388-3397, doi:10.1073/pnas.1305275110 (2013).

5 Vihervaara, A. *et al.* Transcriptional response to stress is pre-wired by promoter and enhancer architecture. *Nat Commun* **8**, 255, doi:10.1038/s41467-017-00151-0 (2017).

6 Consortium, E. P. An integrated encyclopedia of DNA elements in the human genome. *Nature* **489**, 57-74, doi:10.1038/nature11247 (2012).

7 Baranello, L. *et al.* RNA Polymerase II Regulates Topoisomerase 1 Activity to Favor Efficient Transcription. *Cell* **165**, 357-371, doi:10.1016/j.cell.2016.02.036 (2016).

8 Rao, S. S. *et al.* A 3D map of the human genome at kilobase resolution reveals principles of chromatin looping. *Cell* **159**, 1665-1680, doi:10.1016/j.cell.2014.11.021 (2014).

9 Ray, J. *et al.* Chromatin conformation remains stable upon extensive transcriptional changes driven by heat shock. *bioRxiv*, 527838, doi:10.1101/527838 (2019).

10 Teves, S. S. & Henikoff, S. Heat shock reduces stalled RNA polymerase II and nucleosome turnover genome-wide. *Genes Dev* **25**, 2387-2397, doi:10.1101/gad.178079.111 (2011).

11 Brown, S. A. & Kingston, R. E. Disruption of downstream chromatin directed by a transcriptional activator. *Genes Dev* **11**, 3116-3121 (1997).

12 Velichko, A. K., Petrova, N. V., Razin, S. V. & Kantidze, O. L. Mechanism of heat stress-induced cellular senescence elucidates the exclusive vulnerability of early S-phase cells to mild genotoxic stress. *Nucleic Acids Res* **43**, 6309-6320, doi:10.1093/nar/gkv573 (2015).

13 Hafner, M. *et al.* Transcriptome-wide identification of RNA-binding protein and microRNA target sites by PAR-CLIP. *Cell* **141**, 129-141, doi:10.1016/j.cell.2010.03.009 (2010).

14 Lebedeva, S. *et al.* Transcriptome-wide analysis of regulatory interactions of the RNA-binding protein HuR. *Mol Cell* **43**, 340-352, doi:10.1016/j.molcel.2011.06.008 (2011).

15 Llobet-Navas, D. *et al.* The microRNA 424/503 cluster reduces CDC25A expression during cell cycle arrest imposed by transforming growth factor beta in mammary epithelial cells. *Mol Cell Biol* **34**, 4216-4231, doi:10.1128/MCB.00611-14 (2014).

16 Farazi, T. A. *et al.* Identification of distinct miRNA target regulation between breast cancer molecular subtypes using AGO2-PAR-CLIP and patient datasets. *Genome Biol* **15**, R9, doi:10.1186/gb-2014-15-1-r9 (2014).

17 Schlackow, M. *et al.* Distinctive Patterns of Transcription and RNA Processing for Human lincRNAs. *Mol Cell* **65**, 25-38, doi:10.1016/j.molcel.2016.11.029 (2017).

18 Ehrensberger, A. H., Kelly, G. P. & Svejstrup, J. Q. Mechanistic interpretation of promoter-proximal peaks and RNAPII density maps. *Cell* **154**, 713-715, doi:10.1016/j.cell.2013.07.032 (2013).

19 Core, L. J. *et al.* Analysis of nascent RNA identifies a unified architecture of initiation regions at mammalian promoters and enhancers. *Nat Genet* **46**, 1311-1320, doi:10.1038/ng.3142 (2014).

20 Zacher, B. *et al.* Accurate Promoter and Enhancer Identification in 127 ENCODE and Roadmap Epigenomics Cell Types and Tissues by GenoSTAN. *PLoS One* **12**, e0169249, doi:10.1371/journal.pone.0169249 (2017).

21 Halfon, M. S. Studying Transcriptional Enhancers: The Founder Fallacy, Validation Creep, and Other Biases. *Trends Genet* **35**, 93-103, doi:10.1016/j.tig.2018.11.004 (2019).

22 Calo, E. & Wysocka, J. Modification of enhancer chromatin: what, how, and why? *Mol Cell* **49**, 825-837, doi:10.1016/j.molcel.2013.01.038 (2013).

23 Stasevich, T. J. *et al.* Regulation of RNA polymerase II activation by histone acetylation in single living cells. *Nature* **516**, 272-275, doi:10.1038/nature13714 (2014).

24 Nojima, T. *et al.* Mammalian NET-Seq Reveals Genome-wide Nascent Transcription Coupled to RNA Processing. *Cell* **161**, 526-540, doi:10.1016/j.cell.2015.03.027 (2015).

25 Nojima, T., Gomes, T., Carmo-Fonseca, M. & Proudfoot, N. J. Mammalian NET-seq analysis defines nascent RNA profiles and associated RNA processing genome-wide. *Nat Protoc* **11**, 413-428, doi:10.1038/nprot.2016.012 (2016).

26 Shalgi, R. *et al.* Widespread regulation of translation by elongation pausing in heat shock. *Mol Cell* **49**, 439-452, doi:10.1016/j.molcel.2012.11.028 (2013).

REVIEWERS' COMMENTS:

Reviewer #1 (Remarks to the Author):

We fully agree with the authors' written responses to the reviewers. In particular, the methods used here are able to distinguish transcription kinetics from steady-state polymerase occupancy, while some published papers have critically failed to make this important distinction.

We'd like to stress the importance of the point the authors' make about occupancy in their response to reviewer 2, and that it is the combination of polymerase occupancy and RNA synthesis rate measurements that allow the authors to make reasonable estimates of important kinetic parameters including productive initiation frequency. The critical complementarity of these two methods can be observed in the striking case of the pause peaks in response figure 4: strong mNET-seq peaks occur in the presence of low TT-seq signal, indicating that high occupancy there is caused by long residence times.

We'd also like to stress the rigor with which the authors addressed critical opportunities for bias in the utilized methods, as is found in the methods section and as they have again summarized in their responses to reviewer 2. This level of rigor is frequently lacking in the published literature.

The authors did an admirable job in addressing reviewer concerns, and we feel the paper has been strengthened and should be approved for publication.
John Lis & Michael DeBerardine

Reviewer #2 (Remarks to the Author):

This revised paper is certainly much improved over the original version. However, it remains my concern that the bioinformatic/mathematical analysis while sophisticated still makes several key assumptions that may not necessarily be valid.

1) It is assumed that TT-seq gives an unambiguous measure of Pol initiation. However, TT-seq must be a measure of Pol II elongation as the thioU analogue has to be incorporated during transcription elongation.
2) It is becoming increasingly likely that a lot of initiated Pol II is subject to premature termination: see Krebs et al. PMID: 28735898, Steurer et al. PMID: 29632207 Erickson et al. PMID: 30150253. This could significantly affect the bioinformatic calculations in this study.
3) I suspect that these assumptions may explain why on page 5 different effects are observed for lincRNAs than previously measured by promoter escape indexes based on mNET-seq (Schlackow et al 2017).

I feel that these issues still need to be addressed in the discussion of these data and that in effect the conclusions from this "multi-omic" analysis need to be appropriately qualified.
Actually, the word multi-omic is inaccurate and should really be multi-transcriptomic?

<u>Point-by-point responses to reviewers' comments</u>

Nature Communication research article NCOMMS-19-02193-T

The pause-initiation limit restricts transcription activation in human cells

by Saskia Gressel, Björn Schwalb, and Patrick Cramer

Responses are in italics

We thank both reviewers for their feedback on the manuscript. We are very thankful to reviewers #1 who are world leading experts in the pausing field for the in-depth assessment and support of the data analysis and kinetic modeling we presented in our manuscript. In addition, we have made every effort to respond to reviewer #2 concerns, and in so doing, we believe we have addressed her/his criticisms.

Reviewer #1:

We fully agree with the authors' written responses to the reviewers. In particular, the methods used here are able to distinguish transcription kinetics from steady-state polymerase occupancy, while some published papers have critically failed to make this important distinction.

We'd like to stress the importance of the point the authors' make about occupancy in their response to reviewer 2, and that it is the combination of polymerase occupancy and RNA synthesis rate measurements that allow the authors to make reasonable estimates of important kinetic parameters including productive initiation frequency. The critical complementarity of these two methods can be observed in the striking case of the pause peaks in response figure 4: strong mNET-seq peaks occur in the presence of low TT-seq signal, indicating that high occupancy there is caused by long residence times.

We'd also like to stress the rigor with which the authors addressed critical opportunities for bias in the utilized methods, as is found in the methods section and as they have again summarized in their responses to reviewer 2. This level of rigor is frequently lacking in the published literature.

The authors did an admirable job in addressing reviewer concerns, and we feel the paper has been strengthened and should be approved for publication.
John Lis & Michael DeBerardine

Thank you for your thorough and diligent assessment of our manuscript. It is highly appreciated.

Reviewer #2:

This revised paper is certainly much improved over the original version.

Thank you.

However, it remains my concern that the bioinformatic/mathematical analysis while sophisticated still makes several key assumptions that may not necessarily be valid.

Our kinetic modeling is built on only two assumptions that are both reasonable and well established: (i) polymerase occupancy depends on the number of polymerases and their speed (Ehrensberger et al. Cell 2013), and (ii) the spike-in normalized TT-seq signal in the exons is proportional to the productive initiation frequency (Schwalb et al. Science 2016; Gressel et al. eLife 2017). As a consequence, the combination of TT-seq and mNET-seq (multi-omics approach) allows to estimate productive initiation frequency and pause duration genome-wide.

Below, we describe again the reasoning and mathematics behind these two assumptions in detail below, and hope this clarifies the concern.

1) It is assumed that TT-seq gives an unambiguous measure of Pol initiation. However, TT-seq must be a measure of Pol II elongation as the thioU analogue has to be incorporated during transcription elongation.

This is a misunderstanding. The TT-seq signal does not provide a measure of elongation or its velocity in bulk cell populations. Rather, its signal is proportional to the number of polymerases passing over a specific genomic location per time. In order to introduce the reviewer to the relevant information needed to understand the next paragraphs we briefly revisit the effects of elongation velocity on the TT-seq and the mNET-seq readout.

A slow-moving polymerase will produce less transcriptional output in the same time as a fast moving one. This corresponds to the 4sU-labeled RNA fragments captured by our TT-seq method. In contrast, a fast-moving polymerase is much less likely to be encountered at a given position than the slow moving one (Ehrensberger et al. 2013; ***Response Figure 6 a****). This is owned to the fact that given its velocity the polymerase has various residence times which result in different (average) spacing between polymerases along the transcription unit (TU). The average polymerase distribution is captured by mNET-seq.*

In TT-seq, however, these two effects exactly compensate each other. TT-seq therefore yields a coverage readout of the same height throughout the gene body, given that there is no polymerase drop-off. Regression analysis shows that only 4% of polymerases drop off over a size of 100 kbp and render this effect negligible. Thus, TT-seq can be used to infer productive initiation frequencies, as these are reflected in the height of the signal in the exons of a TU (the height of the signal in the introns might be influenced by co-transcriptional splicing).

Mathematically speaking, the TT-seq signal, tb, corresponds to the productive initiation frequency I [cell^{-1}min^{-1}]. The reason for this is that the TT-seq signal at each transcribed position x is the number of polymerases observed at this position $p(x) = \frac{I}{v(x)}$ *times their transcriptional output at this position* $v(x) * t$ *(see* ***Response Figure 6 b****). As shown in* ***Response Figure 6 b****, this exactly cancels out the elongation velocity* $v(x)$ *and thus, allows to derive the productive initiation frequency I. Given a labeling duration t=5 [min] and length L of the TU, this results in the following equation:*

$$tb = \frac{I}{v(x)} * v(x) * t * L = I * t * L$$

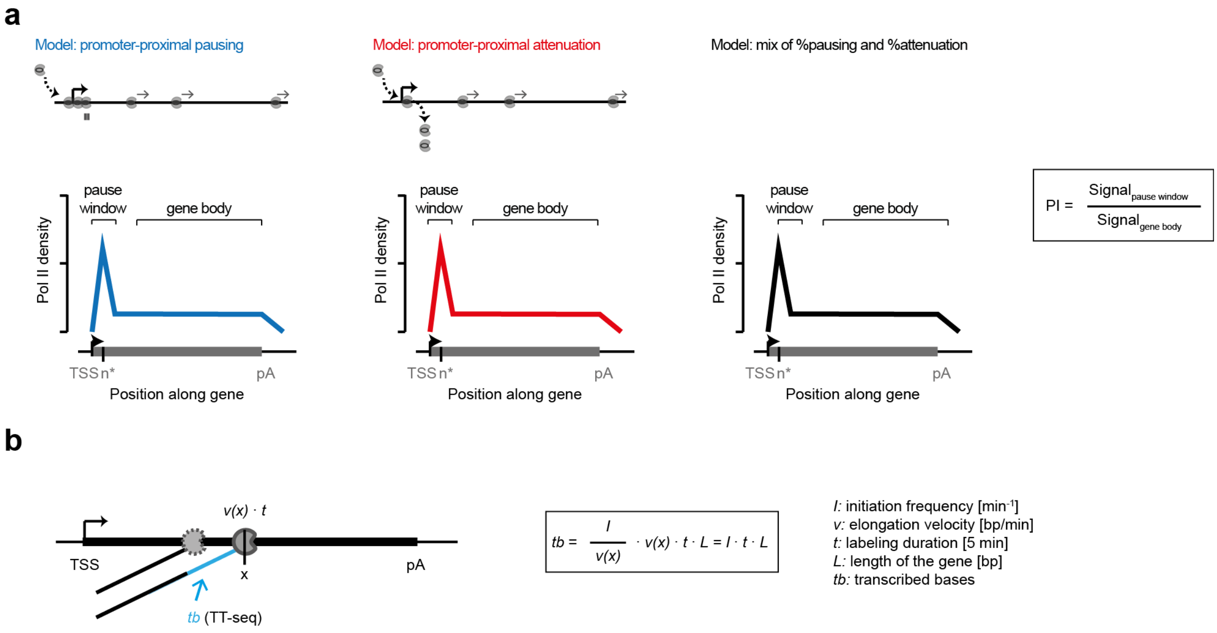

Response Figure 6. ***Only multi-omics allows kinetic analysis.***
(a) Models (top panel) and metagene plots (bottom panel) for promoter proximal peaks. Figure is adapted from Ehrensberger et al. 2013. The pause index (PI) is the ratio of Pol II density in the pause window to the gene body (Muse et al. 2007; Zeitlinger et al. 2007). (b) The TT-seq signal does not provide a measure of elongation or its velocity in bulk cell populations. Instead, TT-seq corresponds to the transcribed bases (tb) during a 5 min labeling pulse (t).

2) It is becoming increasingly likely that a lot of initiated Pol II is subject to premature termination: see Krebs et al. PMID: 28735898, Steurer et al. PMID: 29632207 Erickson et al. PMID: 30150253. This could significantly affect the bioinformatic calculations in this study.

As pointed out above, our model does depend on only two assumptions: occupancy is a mixture of number of polymerases and their speed, and TT-seq measures productive initiation frequency. However, our model is independent of the exact mechanism at the promoter-proximal pause site, may it be pausing or premature termination - and, we acknowledged the possibility of premature termination (attenuation) in our submitted manuscript (lines 648 - 652). The pause duration d obtained in our multi-omics approach reflects the effective pause between two initiation events that successfully lead to productive elongation of a transcript and thus, the relevant transcriptional outcome important for cellular function and health. In the revised version, we added an additional sentence to make it even clearer (lines 652 - 653). In summary, we emphasize again that the events upstream of the pause site are irrelevant to our model and its conclusions. We do not say that it is not possible that a fraction of polymerases terminates at the pause site – in the contrary, by using the productive initiation frequency we actually acknowledge that this can occur.

Promoter-proximal events are highly complicated processes, which we do not presume to describe in its entirety, and we stated so in the manuscript. However, as stated before, to date, no method is available to distinguish attenuation from long pause durations. Additionally, none of the Pol II occupancy methods (incl. mNET-seq), not even short capped RNA assays are able to distinguish the frequency of these events. In bulk cell populations, short capped RNA measurements are as well biased by the residence time of the polymerases since the nascent RNA associated with the elongation complex is longer protected (and thus, measurable) for loci

*with longer pause durations (**Response Figure 6 c-d**). Taken together, it is technically challenging to assess the frequency of these events, and it will be a crucial task for future methods development to quantify the extent of each in order to define the contribution to transcription regulation.*

We carefully assessed, if the papers cited by the reviewer have the potential to infer premature termination in vivo. However, none of them provides definite proof for attenuation, as explained below.

Steurer et al. study the kinetics of Pol II tagged with GFP by photobleaching and measure the rates of fluorescence recovery. However, this visualization cannot distinguish the different complexes of Pol II (e.g. not bound to DNA, promoter or gene body associated). Most importantly, they cannot distinguish between polymerases that terminate or those that may proceed into productive elongation. Only by the addition of several drugs and computational modelling the authors infer attenuation levels, which is perturbing and thus, questionable.

Krebs et al. performed their methylation footprint assays using a 30 min enzymatic treatment at physiological temperature during which Pol II could very likely escape promoter-proximal pausing given the average residing time at the promoter-proximal pause site (Henriques et al. 2013; Jonkers et al. 2014; Zeitlinger et al. 2017; Gressel et al. 2017).

Erickson et al. perform ChIP-seq assays in the presence of high salt (200-500 mM NaCl), and this might prevent factors to bind which under physiological conditions stabilize the paused Pol II complex (e.g. NELF is known to be salt sensitive). In addition, Erickson et al. shown DRB-ChIP-seq only for short TUs (<10 kb) which could be cleared by elongating polymerases within 10 min of release (assuming an average elongation velocity of 2.5 kb/min (Danko et al. 2013; Saponaro et al. 2014; Fuchs et al. 2014; Jonkers et al. 2014; Veloso et al. 2014; Gressel et al. 2017)).

Taken together, we agree that there are active debates about promoter-proximal pausing versus attenuation (for a recent view see Core and Adelman 2019), but this is irrelevant to our model and its conclusions. Our model holds true independent of the percentage of attenuation.

3) I suspect that these assumptions may explain why on page 5 different effects are observed for lincRNAs than previously measured by promoter escape indexes based on mNET-seq (Schlackow et al 2017).

*A key point of our paper is to emphasize again that kinetics cannot be inferred from an occupancy read-out. Thus, mNET-seq data alone (Schlackow et al. 2017) cannot inform on differences in pausing of lincRNAs compared to mRNAs. Please refer to the comments from reviewer #1 as well as to our **Supplementary Note 1** and **Supplementary Figure 6**, the original interpretation of polymerase density maps (Ehrensberger et al. 2013), and to our paper introducing the multi-omics approach (Gressel et al. 2017).*

*The readout of polymerase engaged methods, such as mNET-seq, is the polymerase density on DNA. RNA is solely the messenger for mapping polymerase occupancy onto the DNA. These densities are dictated by the elongation velocities of polymerases, i.e. their residence times at certain positions and the resulting likelihood to encounter and measure them. The consequential average spacing between those polymerases on the DNA template is exactly what makes us observe regions that are more or less populated with polymerases, such as promoter proximal regions or the gene body (**Supplementary Figure 6 a-d**).*

I feel that these issues still need to be addressed in the discussion of these data and that in effect the conclusions from this "multi-omic" analysis need to be appropriately qualified.

We went through the text again and made sure it is correctly understood that our results do not exclude the possibility of a fraction of polymerases to terminate pre-maturely (attenuate). We also added a sentence to the discussion (lines 278 - 281) that points out again that we cannot exclude that a fraction of polymerases terminates pre-maturely in the pause window (attenuation). Therefore, we feel that these issues are very well addressed in the current manuscript and would like to keep the text as it is.

Actually, the word multi-omic is inaccurate and should really be multi-transcriptomic?

We would like to keep the term multi-omics because NET-seq is not a transcriptomic technique, but rather a method to map the occupancy of the genome with engaged polymerase. In general, '-omics' techniques produce genome-wide data sets which require statistical and computational efforts to delineate genome-scale behavior. The key feature of a 'multi-omics' approach is that additional insights (here, kinetic parameters) are gained through the integration of '-omics' data sets (here, TT-seq and mNET-seq). Our approach integrates multiple layers of information, i.e. RNA synthesis rates and Pol II protein distribution within the genome, which are required to acquire kinetic parameters. Thus, they provide a description of the RNA levels (transcriptomics) but also of the Pol II protein distribution on DNA (genomics) inferred from the associated RNA molecule. Thus, to stay accurate we would like to keep the wording as it is.
